# Plasma Membrane Factor XIIIA Transglutaminase Activity Regulates Osteoblast Matrix Secretion and Deposition by Affecting Microtubule Dynamics

**DOI:** 10.1371/journal.pone.0015893

**Published:** 2011-01-20

**Authors:** Hadil F. Al-Jallad, Vamsee D. Myneni, Sarah A. Piercy-Kotb, Nicolas Chabot, Amina Mulani, Jeffrey W. Keillor, Mari T. Kaartinen

**Affiliations:** 1 Division of Biomedical Sciences, Faculty of Dentistry, McGill University, Montreal, Quebec, Canada; 2 Department of Chemistry, Faculty of Arts and Science, Université de Montréal, Montreal, Quebec, Canada; 3 Division of Experimental Medicine, Department of Medicine, Faculty of Medicine, McGill University, Montreal, Quebec, Canada; University of South Florida College of Medicine, United States

## Abstract

Transglutaminase activity, arising potentially from transglutaminase 2 (TG2) and Factor XIIIA (FXIIIA), has been linked to osteoblast differentiation where it is required for type I collagen and fibronectin matrix deposition. In this study we have used an irreversible TG-inhibitor to ‘block –and-track’ enzyme(s) targeted during osteoblast differentiation. We show that the irreversible TG-inhibitor is highly potent in inhibiting osteoblast differentiation and mineralization and reduces secretion of both fibronectin and type I collagen and their release from the cell surface. Tracking of the dansyl probe by Western blotting and immunofluorescence microscopy demonstrated that the inhibitor targets plasma membrane-associated FXIIIA. TG2 appears not to contribute to crosslinking activity on the osteoblast surface. Inhibition of FXIIIA with NC9 resulted in defective secretory vesicle delivery to the plasma membrane which was attributable to a disorganized microtubule network and decreased microtubule association with the plasma membrane. NC9 inhibition of FXIIIA resulted in destabilization of microtubules as assessed by cellular Glu-tubulin levels. Furthermore, NC9 blocked modification of Glu-tubulin into 150 kDa high-molecular weight Glu-tubulin form which was specifically localized to the plasma membrane. FXIIIA enzyme and its crosslinking activity were colocalized with plasma membrane-associated tubulin, and thus, it appears that FXIIIA crosslinking activity is directed towards stabilizing the interaction of microtubules with the plasma membrane. Our work provides the first mechanistic cues as to how transglutaminase activity could affect protein secretion and matrix deposition in osteoblasts and suggests a novel function for plasma membrane FXIIIA in microtubule dynamics.

## Introduction

Bone is a highly dynamic connective tissue that is remodelled throughout life by the reciprocal activity of two different bone cell types – osteoclasts that resorb bone and osteoblasts that form new bone. Defective osteoblast activity leads to insufficient bone deposition and contributes to the development of bone degenerative diseases such as osteoporosis [1], [2]. Osteoblasts arise from mesenchymal origins and lineage differentiation is under the master control of Cbfa1 (Runx2) and Osterix transcription factors as well as myriad of signaling pathways [3]–[5]. Mature and fully differentiated osteoblasts deposit bone matrix, of which 90% is collagen type I (COL I) by weight; the remaining 10% is composed of bone matrix noncollagenous proteins and small proteoglycans, many still having unassigned functions [1], [6]. The fibrillar COL I matrix, in addition to being a main structural determinant in bone, also plays a major role in regulating osteoblast activity and it is known to be required for expression of osteoblast markers, such as alkaline phosphatase [7]–[9], which promotes the removal of inhibitory pyrophosphate thus promoting mineral deposition – the end stage of bone formation [10]. COL I synthesis, secretion, assembly and deposition are regulated by a vast and multilevel cellular machinery that includes COL I modifying enzymes (such as prolyl-hydroxylases) that influence COL I stability, COL I folding chaperones, Golgi-to-plasma membrane trafficking of COL I containing secretory vesicles [11]–[13], propeptidases, and matrix residing factors such as fibronectin (FN), the latter acting as a provisional scaffold for COL I deposition and matrix assembly [14], [15]. Appropriate, sequential orchestration of each step is required for elaboration of permanent COL I matrix receptive to mineralization.

We have recently demonstrated that FN and COL I matrix deposition by osteoblasts require transglutaminase (TG) enzyme activity; however, the precise mechanisms of action have remained largely unknown [16]. TGs are a family (currently comprising nine proteins; TG1, TG2, TG3, TG4, TG5, TG6, TG7, Factor XIIIA and inactive erythrocyte TG) of widely distributed enzymes that catalyze a Ca^2+^-dependent acyl-transfer reaction between polypeptide-bound glutamine residues and primary amines, resulting in the formation of a covalent γ-(glutamyl)-ε-lysyl bond (an isopeptide crosslink) between substrate proteins [17]–[20]. This enzymatic reaction is exclusively performed by TGs and can take place at the cell surface and/or in extracellular matrix (ECM) compartments and in cytosol in elevated Ca^2+^ concentration. The active site of TG enzymes is highly conserved throughout the family and across different species and includes a central cysteine residue [19], [20]. TG-reactive glutamine-donor substrates include a vast array of proteins. Some of them are matricellular cell adhesion proteins such as laminin, fibronectin, thrombospondin and osteopontin [21]–[26]; however, intracellular and cytoskeletal substrates have been also been identified [27], [28]. TGs are thought to stabilize cell-matrix adhesion and the matrix itself, by fixing and crosslinking its constituents [18], however, the cellular functions of many TG substrates still remains obscure. We have demonstrated that TG-activity affects FN and COL I matrix formation and have shown that MC3T3-E1 osteoblasts express two TG family members, transglutaminase 2 (TG2) and Factor XIIIA (FXIIIA), both of which are also found in bone *in vivo* [16], [29], [30]. Both TG2 and FXIIIA have long been linked to formation of skeletal elements and have been shown expressed during both intramembraneous and endochondral ossification [29], [31], [32]. TG2, which is the most widely distributed TG enzyme, appears to have a major function on the cell surface in cell adhesion as a FN-binding, co-factor for β1-integrin [33]–[39] and it has been demonstrated that this function may not implicate its crosslinking activity [37], [38], [40]. Cell-surface TG2 promotes integrin clustering and potentiates integrin downstream effects including RhoA activation [41]. TG2 also appears to have a pro-mineralizing effect in chondrocytes and vascular smooth muscle cells [42], [43] and it has been also linked to chondrocyte hypertrophy [44]–[49]. FXIIIA – best known as a blood coagulation cascade transglutaminase [50], [51] – is also found in osteoblasts and in hypertrophic chondrocytes [16], [46], [49], [52]. Indeed, growing evidence indicates that these two enzymes have similar and/or overlapping, but not necessary identical, functions in connective tissue cells.

Although it is evident from our previous work that TG activity regulates osteoblast differentiation and matrix deposition in cell culture [16], the existing *in vivo* data from individual TG2 - and FXIIIA-knockout mice show no immediate bone-related developmental phenotypes [53], [54], [55]. Hence, no specific roles for either of these enzymes have been assigned in bone or in cartilage developmental processes and it has been suggested that TG2 and FXIIIA have either a synergistic function or functional redundancy, and that they can in some cases compensate for each other [52], [56], [57]. Indeed, Tarantino et al. (2009) have recently shown that the observed normal musculoskeletal phenotype of TG2 knockout mice likely arises from the overexpression of FXIIIA in these tissues. Furthermore, it has been shown that TG2 is required for the mobilization of FXIIIA in hypertrophic chondrocytes [49], again indicating that the two enzymes could be in complementary pathways. Despite this potential tight functional link between TG2 and FXIIIA, the two enzymes have very different expression levels and localization patterns in osteoblasts [16]. In osteoblasts TG2 mRNA and protein levels remain constant throughout the differentiation program, and TG2 is found in a punctate pattern on the cell surface [16], which likely represents recycling endosomes where TG2 is found in fibroblasts and endothelial cells [58]. FXIIIA mRNA and protein expression on the other hand are dramatically induced by ascorbic acid [16], which is also known to stimulate the MAP kinase pathway, ERK1/2 signaling and COL I expression [59]. Furthermore, FXIIIA levels continue to increase steadily during differentiation and the enzyme appears in abundance on the cell surface and in the ECM upon ascorbic acid treatment, indicating that FXIIIA could play a major role in osteoblast differentiation and COL I matrix formation [16].

In the present study, we have examined the role and mechanism of action of TG activity during osteoblast differentiation. We sought first to determine which of the two TGs – TG2 and/or FXIIIA – contribute to protein crosslinking activity, and second, to determine where this crosslinking occurs and how it might affect COL I matrix formation. Since accumulating evidence (as outlined above) implies that genetic ablation of these two enzymes can cause compensatory expression of the other enzyme [56] and hence possibly a misrepresentation of function, we have opted for a chemical “block-and-track” approach to reveal activity and to elucidate which enzyme(s) is active during cell differentiation. Our approach makes use of an irreversible TG inhibitor (NC9) that mimics the commonly used TG donor substrate analogue, Cbz-Gln-Gly and bears a dansyl group that allows inhibitor tracking [60]. In this report, we show that NC9 is highly potent in inhibiting osteoblast differentiation and mineralization, where it reacts with and deactivates FXIIIA. TG2 crosslinking activity does not contribute to osteoblast differentiation. Our work also provides the first mechanistic cues as to how transglutaminase activity could affect protein secretion and matrix deposition and suggests a highly novel function for plasma membrane FXIIIA in microtubule dynamics.

## Results

### TG activity regulates collagen deposition and mineralization in osteoblast cultures

**Figure 1:**
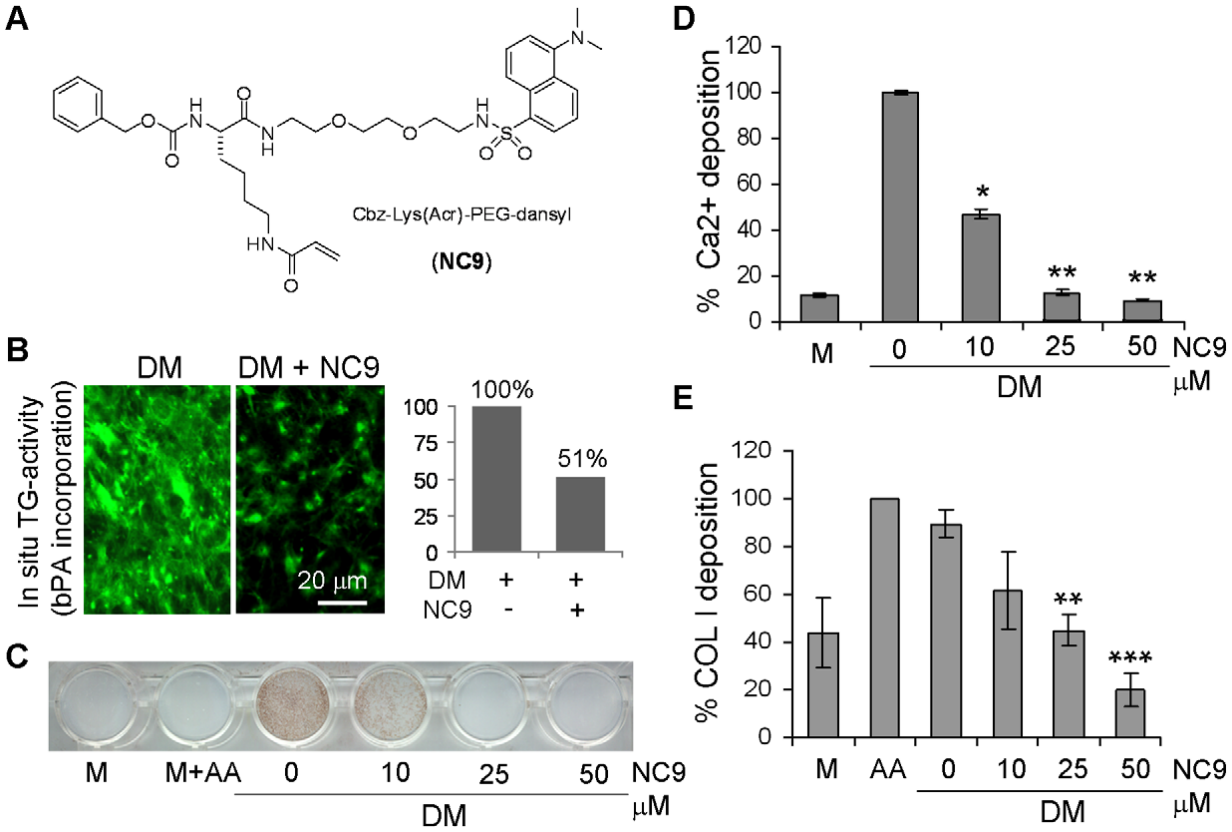
Dose-dependent inhibition of mineralization and COL I deposition in MC3T3-E1 osteoblast cultures by the irreversible TG inhibitor NC9. (A) Structure of NC9. Activity is based on the peptidic scaffold, which provides affinity for the glutamine substrate binding site, and on an electrophilic acryloyl group on the amino acid side-chain that is attacked by the active site cysteine. The inhibitor is thus attached to active site, covalently and irreversibly. (B) *In situ* TG-activity (biotin-pentylamine, bPA, labeling) of the control cultures treated with differentiation medium (DM) alone and with DM and 25 µM NC9. NC9 treatment reduced TG activity in cultures down to 51% of the control as estimated by fluorescence intensity quantification using IMAGE-J software (version 1.37a, National Institutes of Health, USA). (C) Inhibition of mineral deposition with NC9. Mineralization was visualized by von Kossa staining. Complete absence of mineralization was observed in the presence of 25 and 50 µM NC9. (D) Reduction of Ca^2+^ deposition in the NC9-treated cultures. Ca^2+^ deposition in the cell layers was reduced to 46%, 12% and 9% of control with 10, 25 and 50 µM NC9, respectively. (E) COL I deposition in the cultures as assessed by Picrosirius staining. COL I levels were reduced down to 61%, 45% and 19% of the controls with 10, 25 and 50 µM NC9 treatments, respectively. M, medium; DM; differentiation medium (AA+βGP). Error bars represent s.e.m. of two separate experiments done in triplicate.

To investigate the mechanisms by which TG activity regulates osteoblast differentiation and COL I deposition, we have used an irreversible inhibitor to block TG crosslinking activity. This inhibitor, referred to herein as NC9, bears similarity to previously developed irreversible inhibitors designed to mimic the commonly used TG donor substrate Cbz-Gln-Gly [61], [62]. Synthetic schemes are included in the Materials and Methods. As shown in Fig. 1A, the central peptidic scaffold bears a Cbz group, conferring affinity for the glutamine substrate binding site. On the amino acid side-chain it bears an electrophilic acryloyl group that can react with the active-site Cys thiol, blocking it covalently and irreversibly. NC9 also bears a dansyl group, attached to the peptidic moiety via a short PEG spacer, which allows tracking of the site of inhibition and enzymes that react with the inhibitor.

MC3T3-E1 cell differentiation was induced with AA+βGP supplementation to the culture media, referred to herein as differentiation medium (DM). As determined by the MTT cell viability assay, NC9 was nontoxic to the cells at up to 100 µM concentration, and it did not affect general cell growth (data not shown). In the presence of 25 µM NC9, *in situ* TG reactivity with a biotin-pentyl amine probe (bPA) was shown to result in markedly decreased TG-mediated labeling of the cell layers (Fig. 1B). Fluorescence quantification, that was used to estimate TG-activity in cultures, showed a reduction down to 51% of the control value upon NC9 treatment (Fig. 1B). At the highest concentration tested of 50 µM, NC9 blocked mineralization of the cultures, as visualized by von Kossa staining, and reduced Ca^2+^ accumulation in cell-matrix layer to 10% of the control level (Fig. 1C,D). Similarly, COL I deposition, as measured by Picrosirius staining, was reduced significantly to 20% of controls upon 50 µM NC9 treatment (Fig. 1E). Control compound NC10, which was designed to be identical to NC9 but without a reactive warhead side chain had minimal effects on COL I deposition, and was not incorporated into any cellular proteins (Fig. S1).

### TG inhibition blocks COL I secretion and release from the cell surface

**Figure 2:**
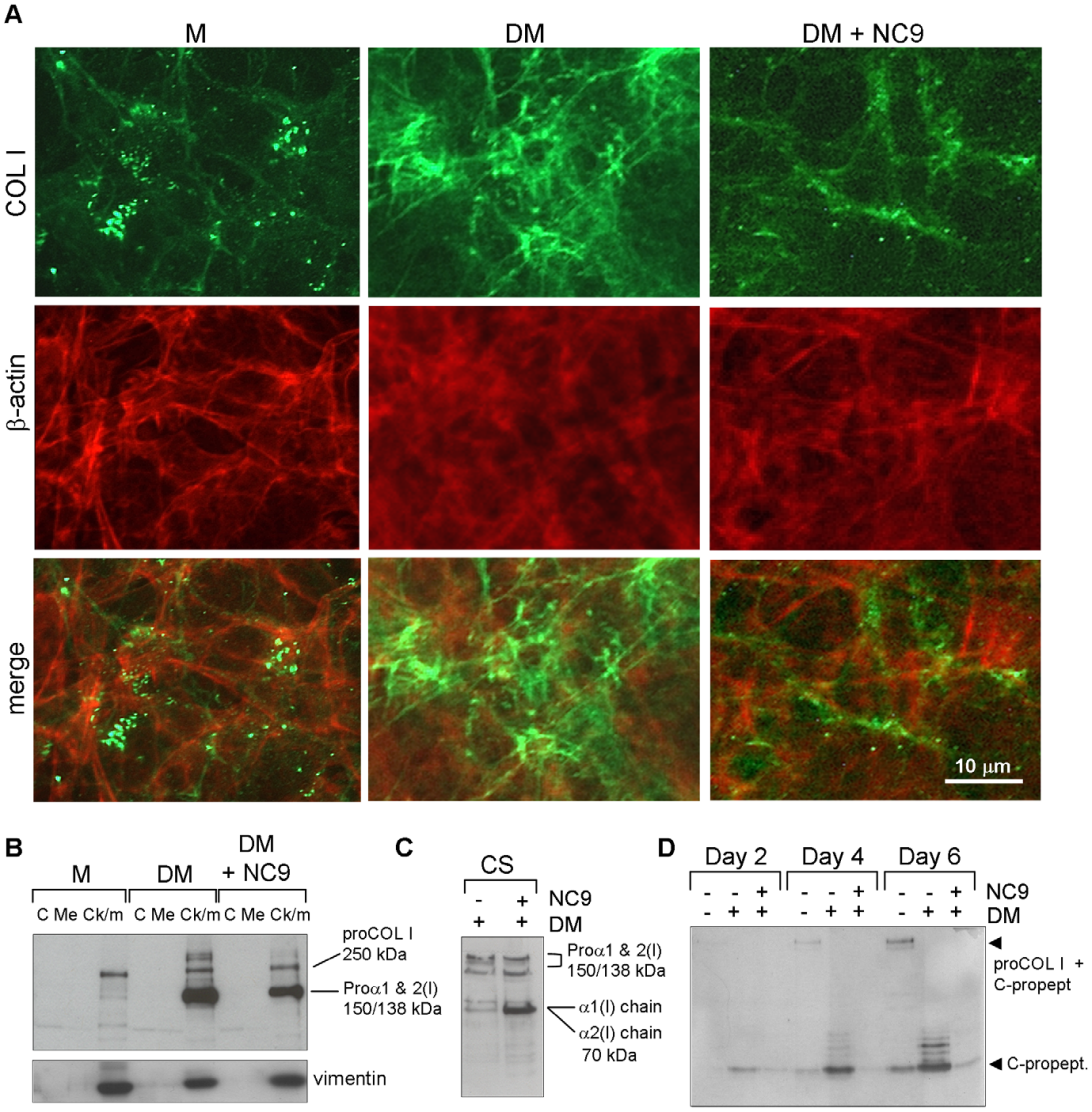
NC9 treatment reduces COL I deposition and secretion. (A) Immunofluorescence staining of COL I (green) in nonpermeabilized control (DM) and NC9-treated cultures. Cytoskeletal actin was visualized with phalloidin-568 (orange). COL I was deposited into a fibrillar network in differentiating cells (with DM) whereas NC9-treated cells had only discontinuous, punctate COL I in the matrix. (B) Subcellular localization (C, cytosolic, Me, membrane; Ck/m, cytoskeleton/matrix) of COL I in NC9-treated and control cells. Vimentin was used as loading control for the cytoskeletal/matrix fraction. Cells treated with NC9 produce COL I although at slightly lower level than differentiating cells. COL I is associated with the cytoskeleton. (C) Cell surface (CS) bound COL I as analyzed from biotinylated cell surface material. NC9-treated cells process some COL I but retain it on the cell surface. (D) COL I assembly and secretion into conditioned medium as analyzed by release of collagen C-terminal propeptide from the tropocollagen (by LF-41 antibody). NC9 treatment reduces COL I secretion and assembly into the matrix.

A detailed investigation of cellular COL I production by immunofluoresence microscopy showed that extracellular COL I levels of the NC9-treated cell cultures were markedly reduced. Fibrils were scant and COL I staining appeared punctate and discontinuous (Fig. 2A). The residual matrix COL I appeared bound to the cells as evident by colocalization of COL I with actin (Fig. 2A). In cells treated with α-MEM medium only, small amounts of COL I were produced (Fig. 2A). Control cells treated with differentiation medium alone deposited normal COL I fibrils. These findings were further supported by Western blot analysis of subcellular fractions (cytosolic, membrane and cytoskeletal/matrix) which showed that NC9-treated cells had begun α1(I) and α2(I) collagen chain production similarly as normal differentiating cells. In both treatments proteins were found in the cytoskeletal-matrix fraction, although slightly lower levels were observed upon NC9-treatment (Fig. 2B). Analysis of COL I levels on the cell surface via biotinylation experiments demonstrated that the NC9 treated cells had externalized and processed some COL I into triple helical format, but COL I mostly cell-associated (individual α-chains at 70 kDa visible under reducing conditions) (Fig. 2C).

To further analyze levels of COL I secretion and processing, we detected COL I C-propeptide levels (LF-41 antibody) [63] in the conditioned medium collected from cells after 2, 4 and 6 days of culture (Fig. 2D). This analysis demonstrated that differentiating cells had expected abundant free, cleaved C-terminal propeptide, but NC9-inhibited cultures had negligible amounts of secreted, free C-terminal propeptide. Collectively, this demonstrates that TG-inhibited cells express and translate COL I mRNA into protein normally, but cannot secrete the protein and/or release collagen to the matrix; i.e., COL I matrix production was arrested at an early secretory and matrix assembly stage.

### Inhibition of TG affects FN deposition and secretion

**Figure 3:**
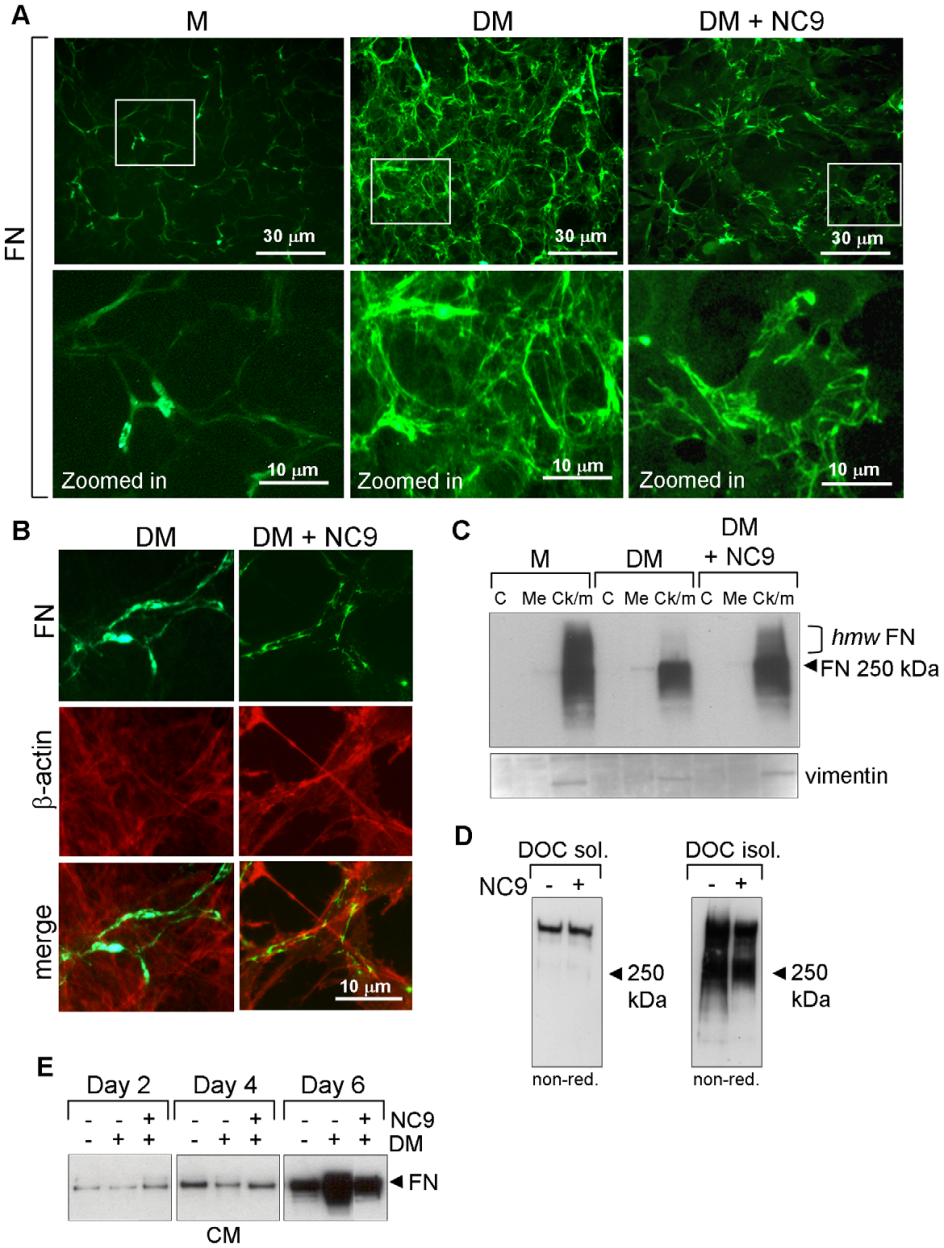
TG inhibition by NC9 reduces FN matrix deposition and secretion by the cells. (A) Immunofluorescence staining of FN matrix (green) in nonpermeabilized control (DM) and NC9-treated cultures. FN matrix assembly was dramatically reduced and FN appeared attached to the cells. Upper panels represent low-magnification images and lower panels are high-magnification images of the boxed areas above. (B) Colocalization of FN (green) and actin cytoskeleton visualized with phalloidin-568 (orange) demonstrating the cellular attachment of FN in NC9-treated cells. No *bone fide* FN fibrils were observed as in control cells. (C) Effect of NC9 on subcellular localization (C, cytosolic, Me, membrane; Ck/m, cytoskeleton/matrix) of FN in NC9 and control cells as analyzed by Western blotting. All cellular FN was found in cytoskeletal fractions in all treatments. Vimentin was used as loading control of the cytoskeletal fraction. Normal, differentiating (with DM) cells appeared to have less high-molecular weight FN (*hmw* FN) suggesting that this processing precedes release of FN to the matrix. (D) Formation of deoxycholate (DOC) soluble and insoluble FN matrix analyzed by nonreducing Western blots. NC9-treatment did not affect the soluble FN matrix, but reduced the formation of DOC-insoluble matrix. (E) FN secretion to conditioned medium (CM) was reduced at day 6 upon NC9 treatment as analyzed by Western blotting.

We have previously reported that TGs can affect FN matrix formation, which begins at a very early stage of osteoblast differentiation process [16]. Also, Mov13 cells which do not express COL I have defective FN matrix deposition [64]. Therefore, we investigated the effects of NC9 on FN matrix. Figure 3A shows that NC9 also reduces FN matrix formation. Closer inspection reveals that cultures treated either with medium alone or with differentiation medium both assemble FN matrix (non-differentiating cultures in lower quantity) whereas NC9-treated cells retain FN at the cell surface (Fig. 3A). Colocalization of FN with cytoskeletal actin shows that FN is indeed associated with the cells and no *bone fide* matrix fibrils are formed (Fig. 3B). The actin cytoskeleton, which participates in FN assembly, was normally formed with inhibitor treatment (Fig. 3B). Comparing FN levels in different subcellular fractions (cytosolic, membrane and cytoskeletal/matrix) (Fig. 3C) showed that slightly more FN was found in the cytoskeleton/matrix fraction in NC9-treated cultures than in normal cells. This cytoskeletal FN appeared to have a higher molecular weight than the FN released from the cell surface of normal cells which could indicate that differentiating cells are releasing FN from cell surface and incorporating it into insoluble matrix, but that NC9 treated cells are not. To investigate the formation of insoluble FN matrix in these cells, we fractionated the cell layers into deoxycholate-soluble and insoluble fractions and analyzed the material by non-reducing Western blotting to visualize FN stabilized via cysteine-bridges. Figure 3D shows that whereas deoxycholate-soluble FN was not changed in differentiating and NC9-treated cells, FN levels in the insoluble fraction were slightly decreased upon NC9 treatment. Secreted FN levels remained similar in all treatments, up to day 4, however, at day 6, inhibitor treatment caused cells to secrete markedly less FN compared to the control cells (Fig. 3E). These results further confirm that TG inhibitor NC9 treated cells are arrested at a very early stage of matrix deposition and/or secretion.

### FXIIIA is the main crosslinking transglutaminase orchestrating matrix assembly

To elucidate the mechanism whereby TGs regulate matrix secretion and deposition, we began by examining which enzyme(s) – TG2 and/or FXIIIA – were able to react with the inhibitor and in which cellular structures inhibition occurred. Inhibitor visualization was achieved by Western blotting and immunofluoresence microscopy using an anti-dansyl antibody to detect the dansyl group of NC9. For identification of the labeled enzyme(s), a new mouse anti-FXIIIA antibody against a region spanning residues 675–688 of mouse FXIIIA was developed. This antibody did not detect FXIIIA in nondifferentiating MC3T3-E1 cells at day 6 (medium only treatment) when FXIIIA mRNA was expressed at very low levels, but detected FXIIIA at day 6 when mRNA had also been induced by DM (see Fig. S2A,B). Similar results were seen with a commercial FXIIIA antibody [16]. Furthermore, this antibody detects full-length FXIIIA in wild type osteoblast extracts, but not in control extracts of FXIIIA-deficient osteoblasts (see Fig. S2C).

**Figure 4:**
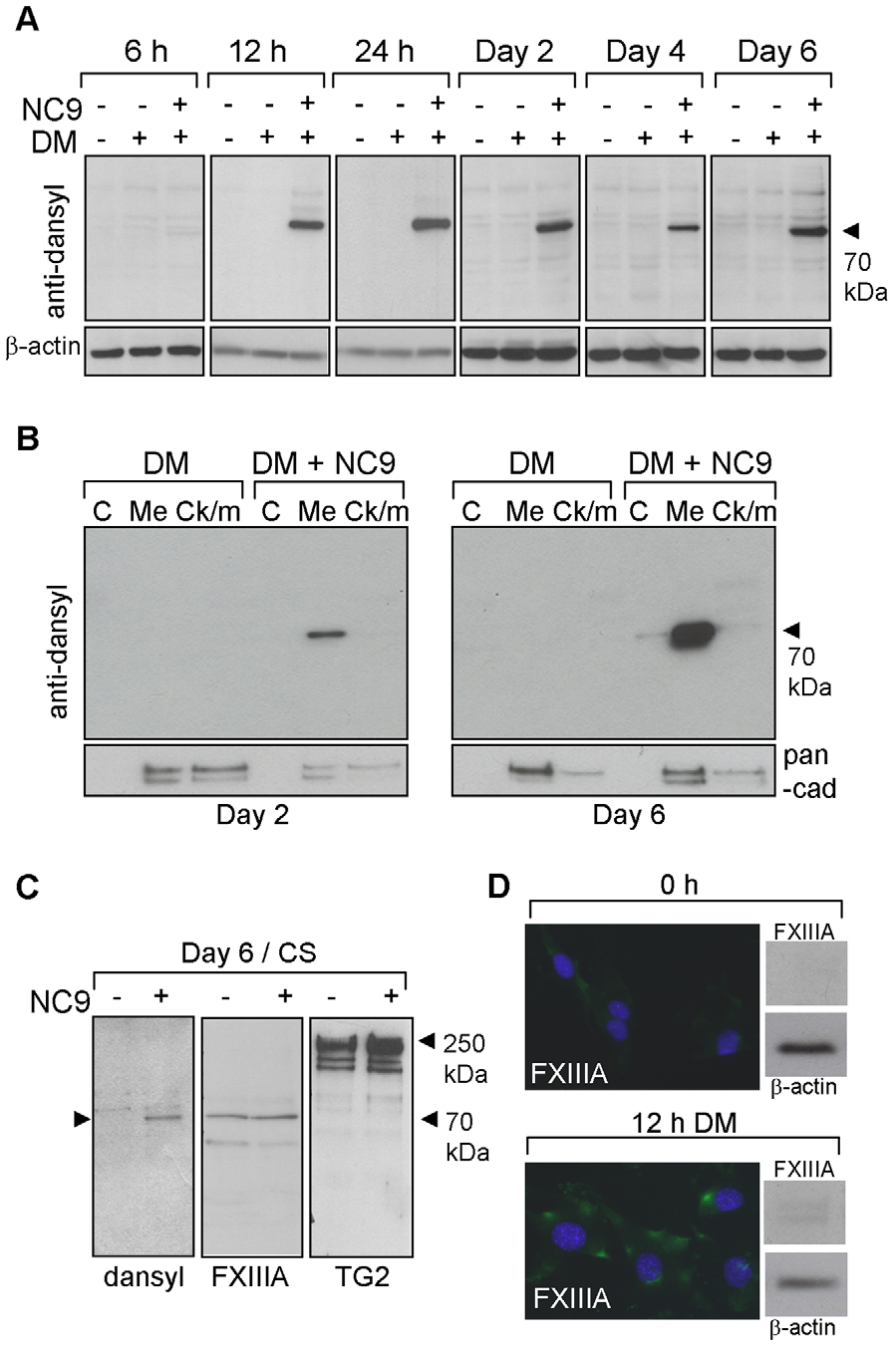
NC9 incorporates into membrane and cell surface-associated FXIIIA. (A) Western blot analysis of NC9 incorporation into osteoblast proteins. Cells were grown in the presence or absence of NC9 from 6 h to 6 days and proteins were extracted and immunoblotted using anti-dansyl antibody. NC9 was incorporated into one protein at 70 kDa from 12 h onwards and levels of labeling remained similar at all time points. No labeling was observed in control cultures (DM) not incubated with NC9. β-Actin was used as loading control. (B) Subcellular localization of dansyl-group revealed that NC9 had labeled FXIIIA in the membrane fraction (Me) and this labeling increased from day 2 to day 6. Pan-cadherin (pan-cad) was used as loading control. (C) Western blot analysis of biotinylated cell surface proteins from NC9-inhibited cultures. NC9, detected by dansyl antibody comigrated with FXIIIA at 70 kDa. Cell surface TG2, which is found in high-molecular weight complexes (≥250 kDa), was not labeled by NC9, indicating that TG2 is not mediating crosslinking during differentiation. (D) Induction of FXIIIA protein expression after 12 h of differentiating treatment (DM) analyzed by immunofluoresence staining of nonpermeabilized cells.

Western blot analysis of control and NC9-treated cultures (Fig. 4A) at time points from 6 h to 6 days after induction of differentiation demonstrated that NC9 is incorporated into one major protein in the cells at ∼70 kDa and was detectable from 12 h onwards – total protein levels remained the same during the entire time course. No labeling was observed in cultures that were not treated with the inhibitor, and no labeling was detected as early as 6 h. Analysis of subcellular fractions showed that NC9 was localized and found mostly in the membrane fraction (Fig. 4B) and that the labeling increased from day 2 to day 6. Since total NC9 labeling appeared not to change (Fig. 4A), it is possible that this represents accumulation of NC9-labelled enzyme on the cell membrane. The observation that NC9 was found in a membrane fraction prompted us to investigate whether NC9 had reacted with FXIIIA or TG2 on the cell surface. For this, we isolated the cell surface proteins via biotin labeling of cells grown with differentiation medium in the presence or absence of NC9. The biotinylation method employed involves a certain level of permeabilization that occurs when the cells are incubated at 4°C; therefore, biotinylation can include also some proteins that are within the plasma membrane [65], [66]. Biotinylated material was detected by dansyl, FXIIIA and TG2 antibodies. As shown in Figure 4C, at the cell surface material the band corresponding to NC9-labeled protein comigrates with FXIIIA band. TG2 was highly abundant on the cell surface in both treatments but *only* found as a high-molecular weight form. Since no NC9 was detected in high molecular weight material, it is likely that TG2 was *not* labeled by NC9. This indicates that cell surface TG2 is not reactive toward NC9 and therefore is inactive and incapable of mediating crosslinking. Since NC9 was found to be incorporated into the cells only from 12 h on, we tested if FXIIIA protein expression was induced between 6 h and 12 h. Immunofluoresence images in Fig. 4D show that at the 6 h time point, cells do not produce FXIIIA but that the protein is present at the 12 h time point. Western blot analysis (Fig. 4D) also shows weak induction of FXIIIA at 12 h time point.

**Figure 5:**
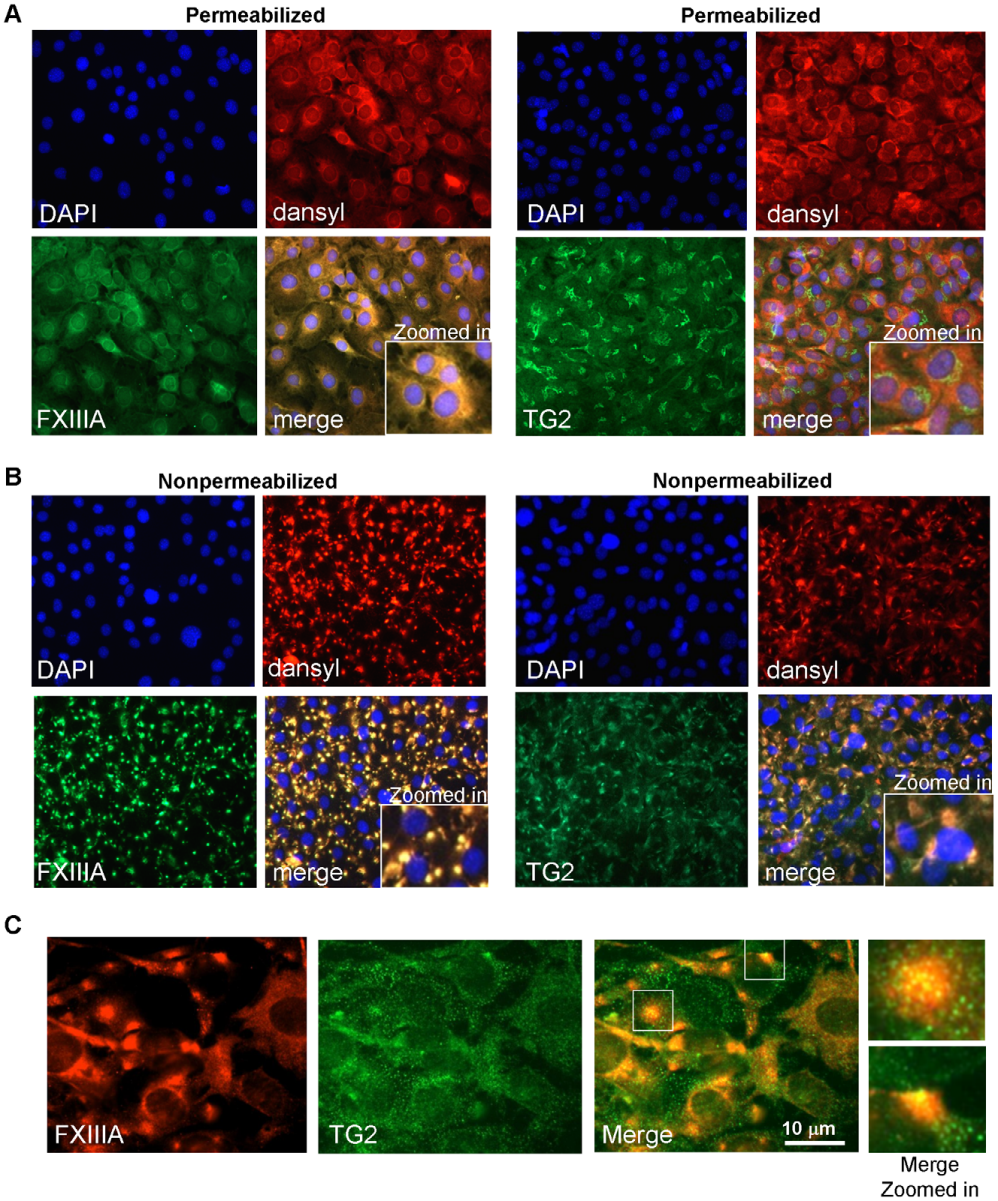
Immunofluorescence colocalization of NC9 with FXIIIA and TG2. (A) Intracellular immunofluoresence colocalization (yellow) of NC9 with FXIIIA and TG2 (both green) shows complete colocalization of the inhibitor with FXIIIA, but no colocalization with TG2. The dansyl group of NC9 was stained with an anti-dansyl antibody followed by anti-rabbit Alexafluor 568 conjugate (orange). (B) Extracellular colocalization (nonpermeabilized cells) of NC9 with FXIIIA and TG2 shows complete colocalization (yellow) of the inhibitor with FXIIIA and limited colocalization with TG2 in a patch-type staining pattern. (C) Colocalization of FXIIIA with TG2 (yellow) showed that these two enzymes are found together on the cell surface – FXIIIA in larger patches and TG2 in small vesicles.

For immunofluorescence microscopy detection of cellular enzymes labeled by NC9, both Triton X-100-permeabilized and nonpermeabilized cells were used to visualize intracellular and cell surface/extracellular dansyl-labeling, respectively. Figure S3 shows that permeabilization allows visualization of cytosolic protein, GAPDH, and nucleus with DAPI and omission of this step blocks cytosolic GAPDH staining but not the staining of nucleus. Detection of permeabilized cells with an anti-dansyl antibody (Fig. 5) showed significant intracellular NC9 labeling that was completely colocalized with FXIIIA, but not with TG2, the latter thought to be inactive and in a closed conformation in the intracellular space at low Ca^2+^ concentration [67]. Since the cytoplasmic extract did not have any covalently incorporated NC9, the intracellular colocalization between FXIIIA and NC9 indicates that the inhibitor interacts with the enzyme, but no crosslinking/catalysis occurs, i.e., FXIIIA enzyme is likely inactive inside the cell. Nonpermeabilized cells showed similar complete colocalization with NC9 and FXIIIA (Fig. 5B), confirming our Western blot results (*vide supra*); i.e., that the cell surface and plasma membrane associated FXIIIA enzyme reacts with NC9. In Fig. 5B TG2 shows some colocalization with NC9; however, since we had confirmed by immunoblotting that TG2 was not labeled by NC9, we hypothesize that the observed partial TG2 and NC9 colocalization derives from TG2 and FXIIIA colocalization on the cell surface. Close examination of nonpermeabilized cells with a higher resolution microscope clearly shows that TG2 and FXIIIA are co-localized and associated on the cell surface (Fig. 5C). TG2 is found in small 1 µm-sized rounded structures that could represent TG2 in endo/exosomes and TG2 clusters with β1-integrin as reported before in endothelial cells [41], [58]. FXIIIA was found to be localized mostly in larger, 5 to 7 µm-diameter rounded patches. TG2 co-localized with FXIIIA in these areas as shown at higher magnification (Fig. 5C, insets). The disappearance of NC9-containing patches with permeabilization using a detergent indicates that they are very sensitive to any changes in plasma membrane integrity and that FXIIIA could be somewhat weakly associated with the plasma membrane.

### NC9 destabilizes microtubules and decreases plasma membrane microtubule levels

Based on all the results above, we hypothesized that FXIIIA crosslinking activity could be part of the secretory machinery and involved in assisting secretory vesicles transport to the plasma membrane to promote matrix protein secretion. Secretory vesicles are transported from the Golgi apparatus to the plasma membrane by motor proteins which move along microtubules (MTs) towards the cell surface where they dock to the cell membrane, fuse and release their contents to the cell surface or to the matrix [12], [68], [69]. The role of MTs in COL I secretion is well established [70]. Hence, we hypothesized that cell surface and plasma membrane associated FXIIIA could regulate secretion by affecting stabilization of MTs and/or assisting MT attachment to the cell cortex and/plasma membrane. Furthermore, MT destabilization has been shown to increase FN interaction with cells, i.e., this being possible explanation why NC9 could also block release of both FN and COL I to the matrix [71].

**Figure 6:**
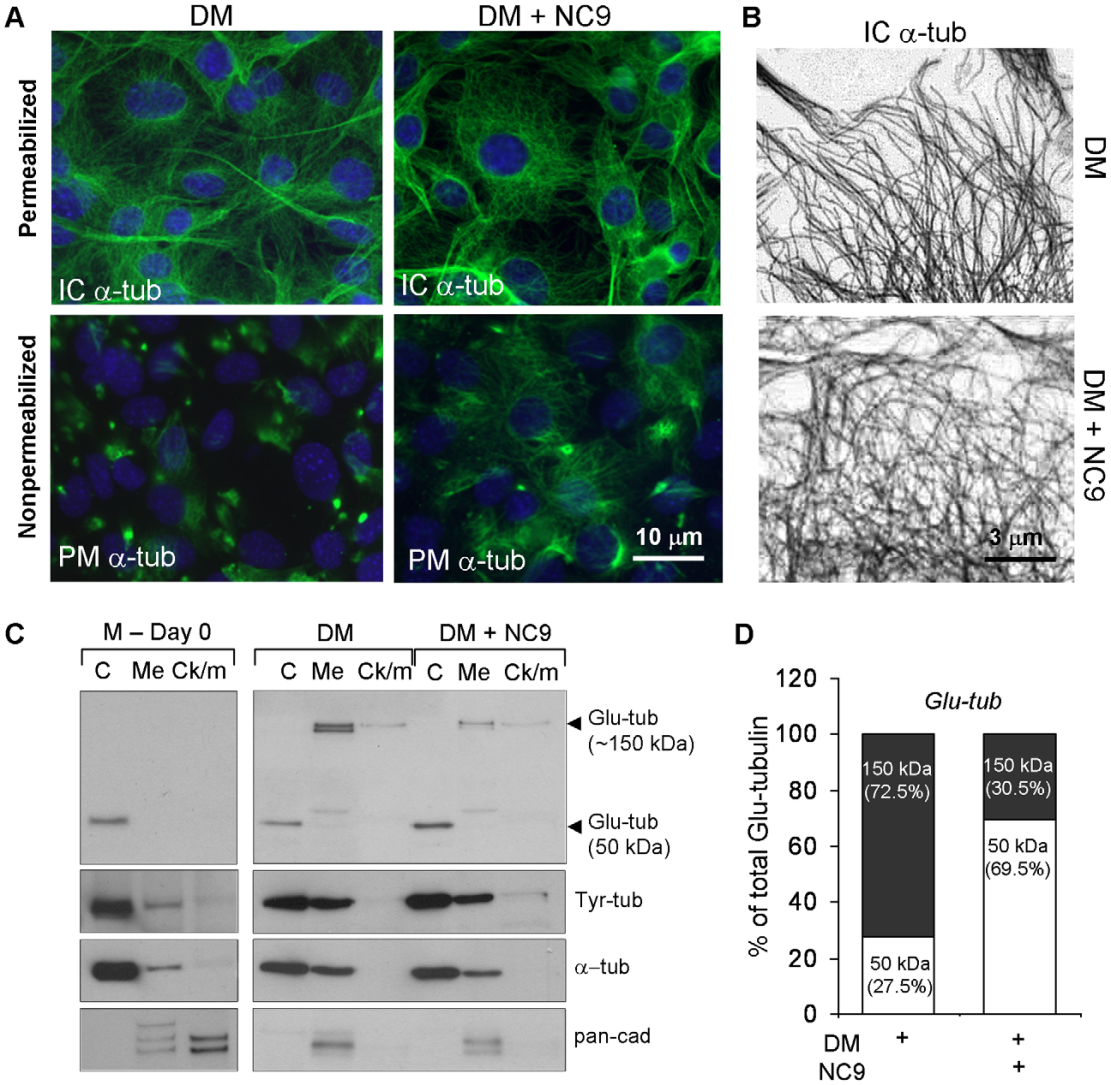
FXIIIA inhibition by NC9 affects microtubule dynamics. (A) Immunofluorescence staining of tubulin (green) and DAPI (blue) in permeabilized and nonpermeabilized cells. Staining under these conditions shows intracellular (IC) tubulin (permeabilized cells) and plasma membrane associated (PM) tubulin (α-tub) (green). Marked difference were found in the distribution of tubulin in the nonpermeabilized cells where tubulin was found in rounded patches in normal cells (DM), but spread along the plasma membrane in NC9-treated cells. (B) High-magnification images of microtubule networks after processing into high-contrast black and white images. FXIIIA inhibited, NC9-treated cells had a more cage-like microtubule network, whereas in control cells microtubules pointed towards the cell surface. (C) Western blot analysis of the levels and subcellular distribution of Glu-tubulin (detyrosinylated and stabilized tubulin) showing that NC9-treated cells have less Glu-tubulin compared to control cells. Glu-tubulin was found in two forms in differentiating cells (DM), as 50 kDa monomer in cytosolic fraction and 150 kDa high molecular weight form which was found in the membrane preparation. Tyr-tubulin and α-tubulin levels were similar with DM and DM+NC9 treatments. Medium only treated cells (M) did not show any 150 kDa Glu-tubulin. (D) Ratio of 150-kDa vs 50-kDa Glu-tubulin in control and NC9-treated cells showing that NC9 decreases the levels of membrane-associated 150-kDa Glu-tubulin and increases the cytosolic 50-kDa Glu-tubulin in the cells. The levels were quantified using Quantity One software and two Glu-tubulin forms are presented as % of total Glu-tubulin and are normalized to α-tubulin levels.

To gain evidence for this, we examined if the MT network is altered in cells that were treated with NC9. Fig. 6A shows that intracellular tubulin levels (in permeabilized cells) initially appear unchanged in both control and NC9-treated cells. However, closer inspection of the MT network, depicted in a high contrast black and white immunofluorescence image (Fig. 6B) shows that control cells have MTs that are directed towards the cell surface, whereas NC9-treated cells show more cage-like MTs whose appearance in this manner has been reported for cells with defective capture to cell cortex [72]. Examination of nonpermeabilized cells (Fig. 6A, lower panels) showed that in control experiment, normal differentiating cells have clusters of plasma membrane-associated tubulin, whereas in NC9-treated cells, the plasma membrane-associated tubulin was spread along the plasma membrane. Medium only treated cells, which do not have FXIIIA on cell surface, also formed these plasma membrane tubulin patches indicating that FXIIIA is not required for their formation, but likely needed for their maintanace during COL I secretion (Fig. S4). Adding NC9 to medium only treated cells that do not produce FXIIIA, had no effect on tubulin patches (Fig. S4). To gain more evidence that in NC9-treated cultures MTs could be destabilized and/or not associated with the plasma membrane, we examined the levels of Glu-(glutamic acid)-tubulin versus Tyr-(tyrosine)-tubulin in subcellular fractions by Western blotting. MTs are stabilized by a detyrosinylation step which exposes Glu-residues in the C-terminus of tubulin such that Glu-tubulin is a marker for MT stability. Fig. 6C shows that in osteoblasts, Glu-tubulin is found in two forms in differentiating and NC9 treated cells - as a 50-kDa monomer and as a 150-kDa form that may represent a Glu-tubulin complex with itself or with another protein. Medium only treated cells, which do not express FXIIIA protein, had only 50 kDa Glu-tubulin (Fig. 6C). NC9 treatment reduced total Glu-tubulin levels (in all cellular fractions added together) to 44% of control, differentiating cells as evaluated after normalization of α-tubulin and Tyr-tubulin. This indicates that FXIIIA is directly or indirectly involved in generation of total Glu-tubulin. Furthermore, the NC9-treated cells were found to have markedly lower levels of the 150-kDa Glu-tubulin and more of the 50-kDa tubulin compared to the control (Fig. 6D) suggesting that changes are occurring as a result of NC9 inhibition of FXIIIA and that that FXIIIA could be involved directly or indirectly in generating these complexes. Moreover, the 150-kDa Glu-tubulin was found mostly in *membrane fraction*, whereas the 50-kDa Glu-tubulin was mostly located in cytosol, indicating that the 150-kDa Glu-tubulin could be associated with the plasma membrane. Tyr-tubulin detection in the same samples showed no changes upon NC9 treatment and it is possible that the Glu-tubulin vs. Tyr-tubulin ratio is so low that minor changes in total Tyr-tubulin levels are simply not observed. Similar observations have been made by others [73]. Interestingly, both Tyr-tubulin and α-tubulin were present in membrane fraction indicating that tubulin can associate with plasma membrane.

**Figure 7:**
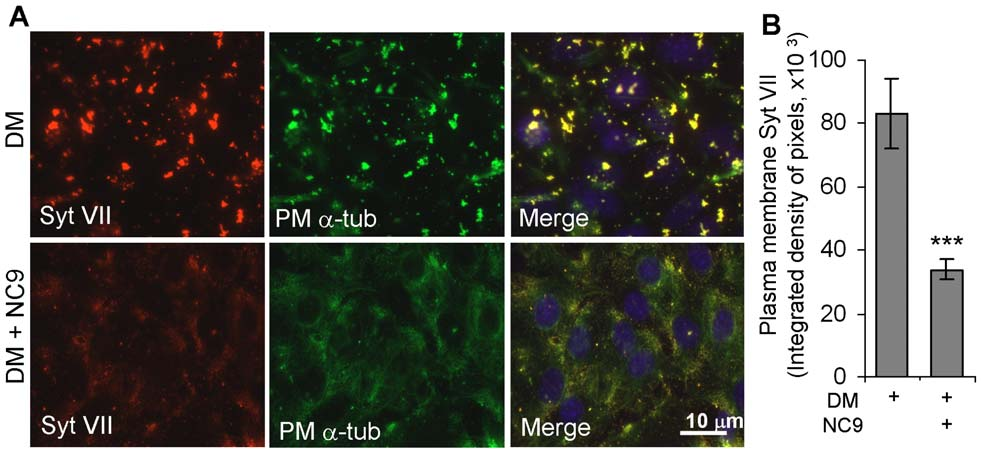
FXIIIA inhibition by NC9 reduces the levels of Synaptotagmin VII, secretory vesicle marker, on the plasma membrane. (A) Immunofluorescence staining of the secretory vesicle marker synaptotagmin VII (Syt VII) and it's colocalization with plasma membrane tubulin in control (DM) and NC9-treated cells. NC9 treatment markedly lowers Syt VII levels and colocalization with tubulin on the plasma membrane. (B) Syt VII levels are significantly lower on the cell surface of NC9-treated cells and decreased down to 29% of controls. Levels were quantified by IMAGE-J software (version 1.37a, National Institutes of Health, USA). Error bars represent s.e.m. of intensity otained from three separate areas in the images.

To determine whether FXIIIA and NC9 could regulate the delivery of secretory vesicles to the cell surface, we investigated the levels of synaptotagmin VII (Syt VII), a secretory vesicle marker on the cell surface. Syt VII was recently demonstrated to be critical for osteoblast function and matrix deposition *in vivo* [74]. Syt VII is involved in docking and fusion of vesicles with the plasma membrane where it participates in formation of the SNARE complex [75]. Synaptotagmin becomes part of the cell membrane during successful exocytosis and portions of the protein protrude the plasma membrane and are detectable on the cell surface of secretory cells from the matrix side [76]. Figure 7A and B shows that Syt VII levels on the plasma membrane and cell surface are significantly lower in NC9 treated cells and decreased down to 29% of controls as measured by immunofluoresence quantification (Fig. 7B). Furthermore, in control cells Syt VII is colocalized with plasma membrane tubulin in patches on the cell surface, whereas in NC9 treated cells have none of this colocalization (Fig. 7A). These results confirm that secretory process is defective and linked to defective microtubule association with plasma membrane. These results also indicate that the observed tubulin patches are linked to the secretory process.

**Figure 8:**
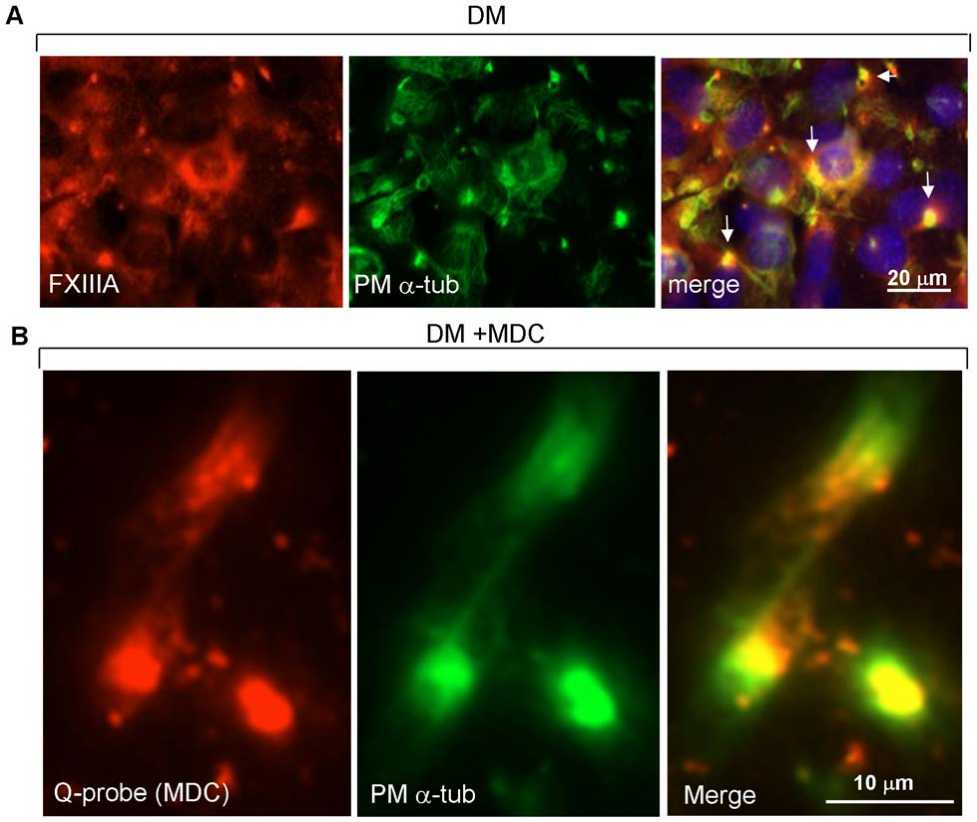
FXIIIA protein and enzyme activity co-localizes with plasma membrane associated tubulin. (A) Immunofluoresence colocalization (yellow) of FXIIIA (orange) and α-tubulin (green) in normal differentiating (DM) osteoblasts. Colocalization (white arrows) is found in rounded patches. (B) *In situ* FXIIIA crosslinking activity is revealed by growing the cells in the presence of Q-probe monodansyl cadaverine (MDC) which incorporates into transglutaminase reactive Q-residues in substrate proteins in the presence of a transglutaminase activity. Dansyl-probe was detected using anti-dansyl antibody (orange) and was found in rounded 5 to 7-µm patches and co-localized (in yellow) with plasma membrane α-tubulin (PM α-tub) (green).

Finally, we examined if FXIIIA and the site of its activity are colocalized with tubulin on the cell surface of normally differentiating cells. Fig. 8A shows that, FXIIIA is found colocalized with plasma membrane-associated tubulin in 5 to 7-µm patches. Enzyme activity and presence of crosslinking was assessed by growing the cells with Q-substrate probe monodansyl cadaverine (MDC), a fluorescent primary amine that mimics a lysine residue in crosslinking reaction and is incorporated into TG-reactive Q-residues in specific proteins that serve as substrates for TGs. We have previously demonstrated that FN is the main Q-donor substrate in osteoblast matrix and accumulates in large quantities in the cells [16], however, many other cellular substrates may exist [27], [28]. As demonstrated in Fig. 8B, immunofluoresence staining shows clear MDC incorporation into the patches and colocalization with tubulin indicating that it could be crosslinked by FXIIIA at these sites.

## Discussion

**Figure 9:**
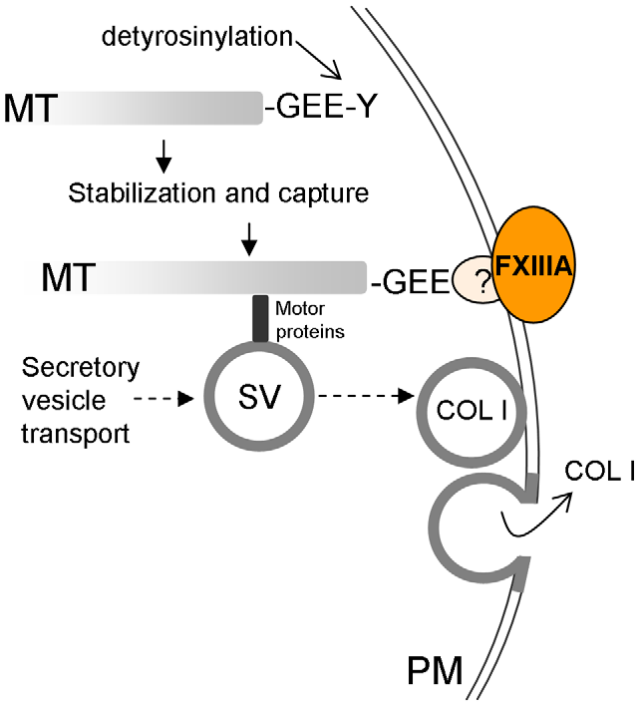
Proposed mechanism whereby FXIIIA crosslinking activity could affect microtubule dynamics and matrix secretion. Microtubules (MTs) are stabilized via a detyrosinylation step and captured to the cell cortex via bridging proteins. Secretory vesicles (SV) are transported along microtubules towards the cell surface where they dock to, and fuse with, the plasma membrane (PM), releasing their cargo to the cell surface or to the extracellular matrix. Plasma membrane associated FXIIIA and it's crosslinking activity may promote the interaction of MTs with plasma membrane which may involve crosslinking of Glu-tubulin to itself to another unidentified bridging protein(s). Precise site of FXIIIA-Glu-tubulin interaction at the cell periphery is unknown.

TG-mediated protein crosslinking has long been associated with matrix formation in physiological events such as bone formation, but also in pathological circumstances such as fibrosis where activity has been mostly linked to TG2 [77], [78]. In this study, we show that during osteoblast differentiation and COL I and FN matrix formation TG2 is inactive and crosslinking activity arises from plasma membrane-associated FXIIIA. Inhibition of FXIIIA results in a disorganized MT network, destabilization of MTs and lower levels of clustered plasma membrane-associated tubulin, as well as decreased secretory vesicle delivery to the plasma membrane indicating that FXIIIA crosslinking activity is directed to stabilize the interaction of MTs with plasma membrane (Fig. 9). Since MT association with plasma membrane is required for the promotion of secretory vesicle (exosome) trafficking and protein delivery to cell surface and secretion to the matrix, our work suggests a mechanism by which FXIIIA can regulate matrix deposition and proposes a novel function for cellular and plasma membrane FXIIIA in MT dynamics.

Although FXIIIA is best known for its role in blood coagulation, where it is present as a pro-enzyme dimer bound to dimeric inhibitory FXIIIB subunits (A_2_B_2_) and where it stabilizes fibrin clots after thrombin activation [50], [51], growing evidence points to additional cellular roles. FXIIIA is expressed by several cell types, including platelets, monocyte-macrophage lineage as well as fibroblasts, chondrocytes and osteoblasts [50], [52], [80] where it has been linked to COL I biosynthesis and deposition in fibroblasts and osteoblasts [16], [81]. The role of cellular FXIIIA in COL I production was also demonstrated in FXIIIA−/− deficient mice that showed decreased COL I production during remodeling and healing after induced myocardial infarction. In these mice COL I levels were not corrected by exogenously administered plasma FXIIIA therapy, indicating that COL I matrix synthesis is regulated by cellular FXIIIA [82]. FXIIIA is also involved in tissue repair and FXIIIA deficiency in humans causes delayed wound closure and healing [83]. Similarly, FXIIIA−/− mice exhibit decreased wound closure and re-epithelization; however, these effects can be corrected by plasma FXIIIA therapy, indicating that wound healing is orchestrated by extracellular FXIIIA [84].

Cellular FXIIIA has been described in cytosol and plasma membrane [80]. Our study demonstrates here that the majority of cellular FXIIIA crosslinking activity in osteoblasts is associated with the plasma membrane, where FXIIIA and its activity are colocalized with tubulin. Plasma membrane-associated FXIIIA has been described, prior to our work, particularly in macrophage-lineage cells [57], [80], [85] where it is colocalized with podosome-like structures and membrane ruffles of migrating cells [57]. Linder and coworkers have described that formation of actin-containing podosomal adhesions is dependent on intact MT network and that MTs are linked to these adhesion structures at macrophage cell cortex [86]. Hence it is possible that FXIIIA could regulate MT dynamics also in macrophages. How FXIIIA associates with the membrane is currently unknown. The observation that NC9-FXIIIA staining disappears upon Triton X-100 permeabilization could indicate that it is weakly associated with outer leaflet of the plasma membrane. Since FXIIIA does not have transmembrane domains, nor a signal peptide that could guide its translocation to the ER-Golgi for modifications such as GPI-anchor insertion, it remains unknown how this protein could be trafficked and inserted to its location. The observation that plasma membrane–associated FXIIIA migrates at ∼70 kDa could indicate that it is either truncated by a protease or modified which could cause different migration. We know from our previous work that these osteoblasts do not generate any FXIIIA splice variants [30]. Also, the precise interaction mechanism between FXIIIA and MTs at the plasma membrane is not clear; however, many reports are available describing plasma membrane-associated tubulin and even cell surface tubulin [87]. Tubulin has been shown to have palmitoylation for membrane insertion in some cell types and it has been demonstrated to be present in lipid rafts and interacting with GM1 and GM3 gangliosides [88].

MTs are an integral part of cellular function and regulate various events from cell motility, to cell shape, to transport and differentiation [79], [89], [90]. MTs are comprised of heterodimeric α- and β-tubulin units that polymerize in a GTP-powered process. MTs emanate from the microtubule organizing center at the centrosome and they grow and shrink in a highly dynamic and rapid polymerization-depolymerization process. MTs are stabilized and protected from depolymerization during various cellular processes including secretion and differentiation [87], [90] and this occurs initially via detyrosinylation that reveals Glu-residues in tubulin (Glu-tubulin). The MTs can be further stabilized by MT-associated proteins (MAPs) and tau which bind along the MTs to decrease depolymerization. MT capture to cell cortex occurs via putative cortical receptors, bridging proteins and MT plus end binding proteins (+TIPs) such as end binding protein-1 (EB1), CLIP170 and CLASP [79], [87], [91]. In our work, we show that in differentiating osteoblasts, Glu-tubulin is present as a 150-kDa membrane-associated form whose levels and membrane association decrease upon NC9-mediated inhibition of FXIIIA. To our knowledge, this higher molecular weight Glu-tubulin form, which could represent a covalent trimeric tubulin complex or a tubulin complex formed with another protein or proteins, has not been described before in mammalian living cellular systems. NC9 also reduces tubulin ‘clustering’ into patches on the plasma membrane and the fact that FXIIIA and it's crosslinking activity is found at sites where tubulin is associated with plasma membrane provides further evidence that such, covalent stabilization, indeed, occurs. Covalent tubulin crosslinking by TGs has been demonstrated before [92], [93] and, interestingly, incubation of monomeric tubulin with TG2 results in high molecular weight tubulin polymers in of 150–160 kDa – similar to what we observe in osteoblasts for Glu-tubulin [94]. The role of this 150 kDa Glu-tubulin can be only speculated at this point, but it may represent an additional stabilization step that could be required for the typical massive and directed COL I secretory process that occurs during *in vitro* bone formation where MTs may need to be ‘locked’ into place at plasma membrane transiently. This ‘locking’ could to provide long term MT stability to allow abundant secretory vesicle trafficking to the plasma membrane via the MT tracks.

In this paper we also describe decreased FN matrix levels upon FXIIIA inhibition by NC9 which could derive from the decreased COL I levels in the cells and/or destabilized MTs [71]. Indeed, our FN staining bears a striking resemblance to FN staining in Mov13 cells which do not produce COL I. In Mov13 cells, most FN is retained on the cell surface [64]. It is not known how COL I regulates FN deposition. Zhang and coworkers [71] have described that destabilizing MTs in fibroblasts by nocodazole or vinblastine increases FN binding (70-kDa fragment) to cell layers and FN assembly on the cell surface. The authors concluded that MT disruption could modulates FN assembly sites and that speculated that the event could be linked to cellular contraction and low cellular GTPase and RhoA levels [71]. The authors also showed that MT stabilization by taxol abolishes FN binding to cells, which could represent promotion of FN release from the cell surface. In other words, MT stabilization could a) promote FN release from cell surface to promote fibrillogenesis, and b) promote COL I secretion and further deposition onto FN fibrils.

Although this study focuses on cellular FXIIIA, we have also published that osteoblasts secrete FXIIIA to matrix and we have further shown that the extracellular FXIIIA activity steadily increases during osteoblast differentiation [16]. Although, we are not addressing the role of secreted FXIIIA in this study, we have shown before that FN in the matrix acts as a major TG substrate during osteoblast differentiation and that it co-localizes with COL I in the matrix suggesting interaction or covalent crosslinking. Hence, it is yet possible that FXIIIA has a further role in stabilizing COL I-FN matrix. TG2 in MC3T3-E1 osteoblasts appears not to be secreted as a full length protein, but as a cleaved fragment which has increased ATPase activity and no crosslinking activity [95], [96]. Therefore, it would be unlikely that TG2 would participate in matrix stabilization. Whether TG2 could influence earlier osteoblast differentiation stages, upstream of FXIIIA function, via its GTPase acivity and/or its role in mediating integrin-FN interaction is currently under investigation. We show in this study that TG2 and FXIIIA are colocalized on the osteoblast surface and links between the functions of the two enzymes are supported by our new findings presented in this paper and recent reports. These include studies from the Belkin group, who have shown that cell surface TG2 binds non-enzymatically to FN and promotes integrin clustering and activates RhoA [41], and studies that show how MTs interact with early focal adhesions and how focal adhesion kinase and RhoA increase MT stability and the levels of Glu-tubulin in fibroblasts attached to FN [73], [97]. This suggests that stable adhesion is required to promote MT stabilization. Hence, the potential synergy between TG2 and FXIIIA could be in the interplay between adhesion and MT network and further stabilization of adhesion via matrix synthesis and assembly.

## Materials and Methods

### Synthesis

The Cbz-protected lysine, and coupling reagents were purchased from GL Biochem; Wang resin was purchased from NovaBiochem. All other reagents were obtained from Sigma-Aldrich. Reactions requiring anhydrous conditions were carried out under a dry nitrogen atmosphere employing conventional benchtop techniques. ^1^H- and ^13^C-NMR spectra were recorded on AMXR400 and AMX300 spectrometers and were referenced to the residual proton or ^13^C signal of the solvent. Mass spectra were determined by FAB+ ionisation on an AutoSpec Q spectrometer. Reactor tubes for solid-phase peptide synthesis were obtained from Supelco. All resins were swelled in dimethylformamide (DMF) and washing steps were performed using CH_2_Cl_2_ (DCM) and DMF (EMD Chemicals). Purification of all peptides was performed using a preparative HPLC method. Mass spectral data (MS, LCMS) were all obtained using two different columns: column A: Gemini C18, 150×4.6 mm, 5 m (Phenomenex, Torrance, CA); column B: Synergi Polar-RP, 150×4.6 mm, 4 m (Phenomenex, Torrance, CA). Crude peptides were purified using a preparative Synergi Polar-RP, 100×21.20 mm (Phenomenex, Torrance, CA) on a Varian (Prep Star) HPLC system.

### ^α^ *N*-Benxzyloxycarbonyl-^ε^ *N*-tert-butoxlycarbonyl-L-lysine (1)

This compound was prepared following our previously published procedure (Keillor, et al. 2008) and spectral data matches that of the commercially available product.

### ^α^ *N*-Carbobenzyloxy-^ε^ *N*-acryloyl-L-lysine (5)

**Figure 10:**
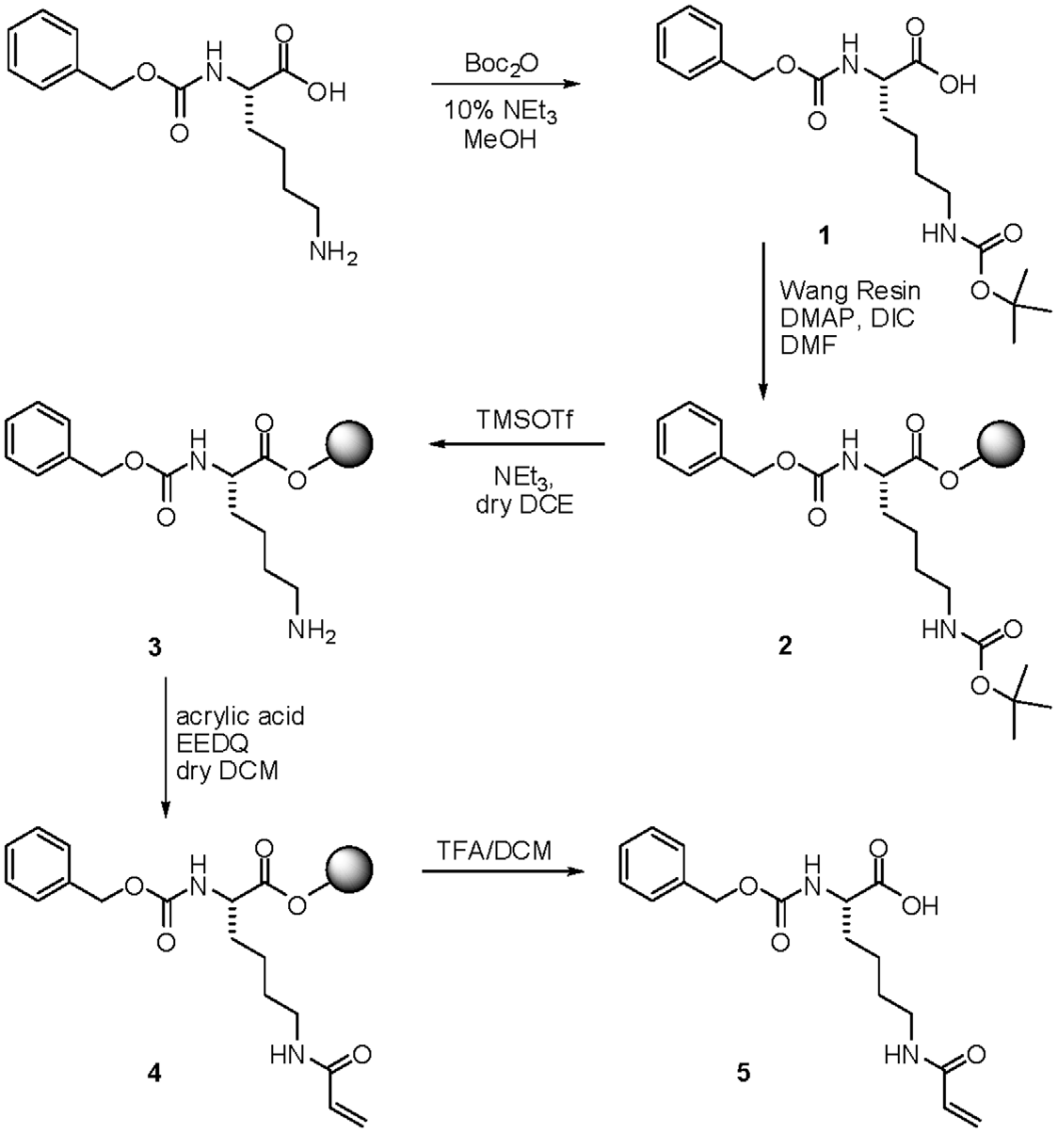
Solid-supported synthesis of synthetic intermediate 5.

The ^α^ *N*-Cbz-protected and ^ε^ *N*-Boc-protected amino acid **1** (5.5 mmol) was coupled to Wang resin (1.1 mmol) using DIC (5.5 mmol) and DMAP (0.11 mmol) (Figure 10). The remaining free resin hydroxyl groups were capped by treating the resin with a mixture of acetic anhydride/pyridine (2∶3) and shaking for 2 h. After washing with DMF (3 times with 10 resin volumes) and DCM (3 times with 10 resin volume), the Boc group was removed as follows: To the reactor containing 1 g of the Wang resin supported Cbz-Boc-lysine (**2**), were added 30 mL of deprotection mixture, freshly prepared from 470 µL TEA (2 eq.), 1.09 mL of TMSOTf (0.2 M) and 28.44 ml of anhydrous DCE. The resin was shaken for 10 min then filtered and washed with 5×5 mL of DCM, 2×5 mL of DIEA 10% in DCM, 3×5 mL of DCE. Deprotection was carried out for another 10 min with a fresh deprotection mixture. The resin was filtered then washed with DCM, DIEA DMF and Et_2_O. Boc removal was verified by positive Kaiser test on a sample of a few beads. To the Wang resin supported Cbz-lysine **3** (1 g) swollen in anhydrous DCM (5 resin volumes) was added acryloyl acid (2.5 eq.) and EEDQ (2.5 eq.). The reaction was shaken for 1 hour, followed by washing with DMF, DCM and ether. The amino acid derivative **4** (Figure 10) was cleaved from the resin (1 g) by incubating with TFA:DCM (1∶1) for 2 hours. The resulting solution was evaporated and cyclohexane was added twice and re-evaporated. A minimal quantity of acetone was added and compound **5** (Figure 10) was precipitated using diethyl ether with a yield of 67%.

^1^H NMR (300 MHz, CDCl_3_) δ 7.25 (m, 5H), 6.97 (d, 1H, *J* = 18 Hz), 6.8 (bs, 1H), 6.13 (m, 2H), 5.53 (d, 1H, *J* = 12 Hz), 5.1 (m, 2H), 4.25 (s, 1H), 3.18 (m, 2H), 1.79 (m, 1H), 1.68 (m, 1H), 1.44 (m, 2H), 1.3 (m, 2H).

^13^C NMR (75 MHz, CDCl_3_) δ 174.5, 166.5, 156.3, 135.8, 130.6, 128.2, 127.9, 127.0, 165.9, 54.0, 39.5, 31.9, 28.1, 22.5.

HRMS (ESI) calcd for C_17_H_22_N_2_O_5_ ([M+H]^+^): 335.1599, found 335.1602.

### (2-(2-dansylaminoethoxy)ethoxy)ethylamine (6)

In a 100-mL round-bottomed flask, dansyl chloride (0.500 g, 1.9 mmol) was added to 30 ml of DCM, along with NEt_3_ (0.26 mL, 1.9 mmol). 1,2-Bis(2-aminoethoxyethane) (2.7 mL, 19 mmol) was added dropwise and the reaction was stirred overnight. Saturated sodium bicarbonate was added to the reaction mixture and the aqueous layer was washed with dichloromethane. The aqueous layer was then acidified and extracted with ethyl acetate. The organic layer was washed with brine, dried over MgSO_4_, filtered and evaporated under reduced pressure to yield a light green oil in 95% isolated yield.

^1^H NMR (300 MHz, CDCl_3_) δ 8.52 (d, 1H, *J* = 8.4 Hz), 8.35 (d, 1H, *J* = 8.7 Hz), 8.23 (d, 1H, *J* = 7.2 Hz), 7.53 (m, 2H), 7.17 (d, 1H, *J* = 7.5 Hz), 3.51 (m, 4H), 3.44 (m, 4H), 3.10 (t, 4H, *J* = 4.8 Hz), 2.88 (s, 6H).

^13^C NMR (75 MHz, CDCl_3_) δ 152.7, 136.1, 131.0, 130.7, 130.5, 130.0, 129.0, 124.0, 119.9, 116.0, 73.3, 71.1, 70.6, 70.3, 46.3, 43.9, 42.2

HRMS (ESI) calcd for C_18_H_28_N_3_O_4_S ([M+H]^+^): 382.1795, found 382.1806.

### ^α^ *N-*Carbobenzyloxy-^ε^ *N*-acryloyl-L-lysine (2-(2-dansylaminoethoxy)ethoxy)ethan-amide (NC9)

**Figure 11:**
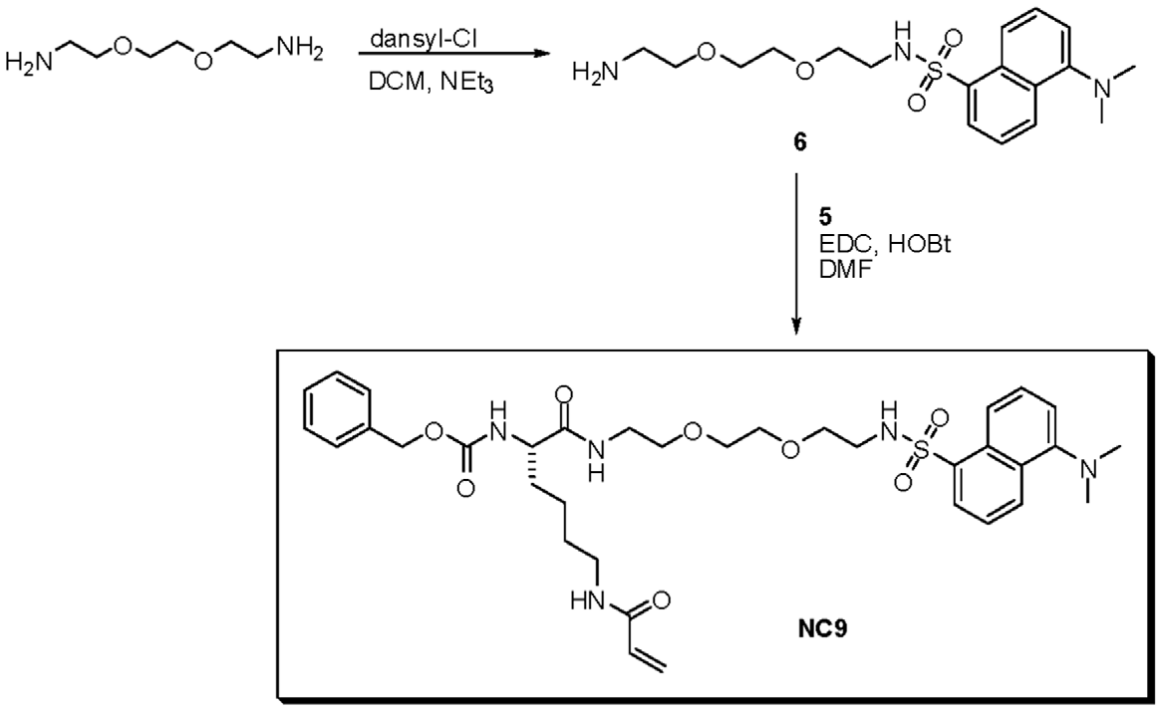
Synthesis of dansyl-labeled probe NC9.

**5** (0.665 g, 1.99 mmol) and **6** (0.910 g, 2.39 mmol) (Figure 11) were combined with HOBt (0.32 g, 2.39 mmol), EDC (0.46 g, 2.39 mmol) and triethylamine (0.3 mL, 2.39 mmol) in a 25-mL round-bottomed flask containing 5 ml of DMF. The reaction mixture was stirred overnight and then poured into 50 mL of H_2_O. An extraction was performed whereby the aqueous phase was washed with dichloromethane and the combined organic phase was then subsequently washed with 1 M HCl, 1 M NaOH and a brine solution. The organic layer was then dried over MgSO_4,_ filtered and the solvent evaporated under reduced pressure. The residue was purified by flash chromatography (96:4 DCM:MeOH) to give the pure product as a greenish-yellow solid in 20% isolated yield.

^1^H NMR (300 MHz, CDCl_3_) δ 8.52 (d, 1H, *J* = 8.5 Hz), 8.29 (d, 1H, *J* = 8.6 Hz), 8.20 (d, 1H, *J* = 6.7 Hz), 7.48 (m, 2H), 7.27 (m, 5H), 7.15 (d, 1H, *J* = 7.5 Hz), 6.95 (m, 1H), 6.19 (m, 2H), 6.02 (m, 1H), 5.78 (d, 1H, *J* = 7.8 Hz), 5.46 (d, 1H, *J* = 9.9 Hz), 5.05 (m, 1H), 5.04 (s, 2H), 4.17 (m, 1H), 3.43 (m, 10H), 3.21 (m, 2H), 3.04 (m, 2H), 2.86 (s, 6H), 1.72 (m, 2H), 1.46 (m, 2H), 1.36 (m, 2H).

^13^C NMR (75 MHz, CDCl_3_) δ 165.4, 161.8, 150.5, 132.1, 131.5, 126.4, 126.0, 125.3, 124.8, 123.8, 123.4, 123.1, 122.3, 121.9, 121.4, 117.5, 115.1, 110.5, 70.2, 69.9, 69.5, 69.3, 67.0, 66.1, 48.5, 44.9, 41.9, 34.7, 33.1, 30.5, 26.9, 26.4, 19.2.

HRMS (ESI) calcd for C_35_H_48_N_5_O_8_S ([M+H]^+^): 698.3218, found 698.3218.

### Cell culture and treatments

MC3T3-E1 pre-osteoblast cells (subclone 14) (a generous gift from Dr. Renny T. Franceschi from the University of Michigan, School of Dentistry) [98] were plated at an initial density of 50,000 cells/cm^2^ for all experiments, except for immunofluorescence microscopy (IFM) where the initial density was 25,000 cells/cm^2^. Cells were grown in minimal essential medium (MEM) containing Earl's salts and nonessential amino acids (Invitrogen; Carlsbad, CA). Medium was supplemented with 10% fetal Bovine Serum (FBS) (PAA Laboratories Inc; Canada), 1% Penicillin-Streptomycin (Invitrogen), 1% L-Glutamine (Invitrogen), and 0.225 mM L-Aspartic acid (Sigma). Cells were grown in a humidified atmosphere in 5% CO_2_. Osteoblast differentiation was initiated 24 hours after plating by supplementing the culture medium with 50 µg/ml ascorbic acid and 10 mM β-glycerophosphate as previously described [9], [16]. This medium is referred to hereafter as differentiation medium (DM). Medium was changed every second day and experimental end points varied from 6 h to 12 days as stated in the text. TG activity was inhibited by using the irreversible inhibitor *N*-α-carbobenzyloxy-*N*-ε-acryloyl-L-lysine (2-(2-dansylaminoethoxy)ethoxy)ethanamide (NC9) (compound 9 in [60]), which was initially used at 10-50 µM concentrations and afterwards at 25 µM only. Toxicity of the inhibitor was tested by analyzing MTT (3-(4,5-dimethyl-2-thiazolyl)-2,5-diphenyl-2H-tetrazolium bromide) (Sigma) incorporation into viable cells. For all osteoblast differentiation assays, cells were incubated with NC9 for 12 days in culture, in all other experiments cells were incubated for 6 days unless otherwise indicated. For TG substrate analysis, cells were grown in the presence of 0.1 mM monodansyl cadaverine (MDC) (Invitrogen, Ca, USA) for 6 days in culture, and then prepared for IFM as described below.

### In situ transglutaminase assay

To study the effect of NC9 on TG activity *in situ*, cells were grown in DM for 6 days in culture, and then treated with 1 mM 5-biotinamide pentylamine (bPA) (Pierce Biotechnology, Rockford, IL) for 12 h before the termination of the experiment. Cells were then prepared for immunofluorescence microscopy (as described below) and stained using anti-biotin antibody (rabbit polyclonal antibody, Rockland, PA, USA) and visualized as described below. Quantification of immunofluorescence intensity was performed using IMAGE-J software (version 1.37a, National Institutes of Health, USA) where the integrated density of pixels (sum of the gray values of each pixel in determined area) of the given image was measured.

### Picrosirius staining and collagen quantification

Collagen production by osteoblasts was assessed by quantification of collagen using Picrosirius Red staining as previously described [99]. Soluble calf skin collagen type I (Sigma) was used for generating standard curves.

### Mineralization and calcium assay

Mineral deposition into the formed collagenous extracellular matrix was visualized by staining the cells at day 12 with 3% silver nitrate (von Kossa staining). Calcium deposition was assessed by measuring Ca^2+^ levels from hydrochloric acid extracts of cell layers using a calcium kit (Diagnostic Chemicals Limited, Oxford, CT).

### Preparation of protein extracts, conditioned medium, subcellular fractions and cell surface proteins

For protein analysis, total cell lysates were prepared by harvesting the cell/matrix layer at the indicated time points using a mild extraction buffer containing 10 mM Tris-HCl pH 7.4, 0.25% sucrose, 0.2% IGPAL, and 1 mM PMSF prepared in phosphate-buffered saline (PBS). Cells extracts were homogenized by ultrasonication directly after extraction and centrifuged at 7500×g for 15 minutes at 4°C. The supernatant was collected and referred to hereafter as total protein extract. Conditioned medium was prepared by changing the cell culture medium to serum-free medium 24 h prior to collection. Subcellular protein fractions where prepared using ProteoExtract Subcellular Proteome Extraction Kit (S-PEK) (EMD Biosciences) following the manufacturer's instructions. Cytoplasmic (C), plasma membranous (Me), cytoskeletal and matrix (Ck/m) preparations were used for this study. Proteins presented on the cell surface were isolated using a cell surface protein isolation kit (Pierce Biotechnology, Rockford, IL) following the manufacturer's instructions. Deoxycholate (DOC) soluble and insoluble material was prepared as described previously in [100] in brief, cell layers were washed with serum free medium, and lysed with DOC lysis buffer (2% deoxycholate, 20 mM Tris-HCl pH 8.8, 2 mM PMSF, 2 mM EDTA, 2 mM iodoacetic acid, and 2 mM *N*-ethylmaleimide). Samples were then centrifuged at 7500 g for 15 min at 10°C and the supernatant was collected and labeled as DOC soluble material. The pellet (containing the DOC insoluble material) was dissolved in DOC insoluble lysis buffer (2% SDS 25 mM Tris-HCl pH 8.8, 2 mM PMSF, 2 mM EDTA, 2 mM iodoacetic acid, and 2 mM *N*-ethylmaleimide). Protein concentrations of all preparations were determined with a bichinchonic acid (BCA) protein assay kit (Pierce Biotechnology, Rockford, IL).

### Western blotting

For Western blot analysis, 10 µg of protein were dissolved in 5 X SDS loading buffer containing β-mercaptoethanol, and boiled for 5 minutes at 100°C. If samples were run in nonreducing condutioned no β-mercaptoethanol was included in sample buffer. Proteins were separated on 10% SDS-polyacrylamide gels under reducing conditions. Subsequently the proteins were transferred onto PVDF membranes, which were blocked in 5% nonfat milk. Membranes were then detected using primary antibodies against the following antigens; COL I (rabbit polyclonal antibody, Millipore), COL I C-terminal pro-peptide (LF-41, rabbit polyclonal antibody, courtesy of Dr. Larry W. Fisher, NIDCR), FN (rabbit polyclonal antibody, Millipore), dansyl-group (rabbit polyclonal antibody, Invitrogen), TG2 (mouse monoclonal antibody, CUB7402/TG100, Thermo scientific), mouse FXIIIA 675-688 peptide sequence (polyclonal antibody, designed and generated by GenScript corporation, USA), synaptotagmin VII (goat polyclonal antibody, Santa Cruz Biotechnology INC., CA, USA), α-tubulin (mouse monoclonal, clone DM1A, Sigma), Glu-tubulin (Rabbit polyclonal antibody, Millipore), Tyr-tubulin (Rabbit polyclonal antibody, Millipore), vimentin (goat polyclonal antibody, Abcam), β-actin (rabbit polyclonal antibody, Sigma) or pan-cadherin (rabbit polyclonal antibody, Abcam) for 2 h at room temperature, followed by brief washing and incubation for 1 h with horseradish peroxidase (HRP-) conjugated anti-mouse (Amersham, Biosciences, Piscataway, NJ), anti-rabbit (Cell Signaling, MA, USA) or anti-goat secondary antibody (Invitrogen, Ca, USA) at room temperature. Reactions were visualized using an ECL detection kit (Amersham Biosciences). Quantification of bands was done with Quantity One software (v 4.5, Bio-Rad).

### Immunofluorescence microscopy

Cells were grown for 6 days on Permanox 8 chamber slides (Lab-Tek Chamber slides, Nalge Nunc International) as indicated above and previously described (Al-Jallad et al., 2006). In short, on day 6 the growth medium was aspirated and the cell layers were rinsed with PBS. Cells were then fixed at room temperature with 3.7% formaldehyde in PBS. For visualization of intracellular proteins, cells were permeabilized with 0.25% Triton X-100 (Sigma) in PBS, whereas for visualization of cell surface and extracellular matrix proteins, permeabilization was omitted. To reduce nonspecific antibody binding, cells were blocked in 2% bovine serum albumin (BSA, Sigma) in PBS for 30 minutes at room temperature. Slides were then stained using antibodies against the following proteins; COL I (rabbit and goat polyclonal antibodies, Millipore), FN (mouse monoclonal antibody, Sigma), dansyl-group (rabbit polyclonal antibody, Invitrogen), TG2 (mouse monoclonal antibody, CUB7402/TG100, Thermo scientific), mouse FXIIIA 675–688 peptide sequence (polyclonal antibody, designed and generated by GenScript corporation, USA), synaptotagmin VII (goat polyclonal antibody, Santa Cruz Biotechnology, Inc) and α-tubulin (mouse monoclonal, clone DM1A, Sigma). Actin cytoskeleton was visualized by Alexa Fluor 594 Phalloidin (Invitrogen) and nuclei were visualized with DAPI (Invitrogen). All antibodies were diluted in 0.1% BSA in PBS and cells were incubated with antibody dilutions as required at room temperature for 2 h. Cells were washed with 0.1% BSA in PBS and incubated with Alexa Fluor secondary antibodies against mouse, rabbit, or goat primary antibodies with wavelength excitations of 488 (green) or 568 (orange) (Invitrogen). Negative controls involved omission of primary antibodies and secondary antibodies. In case of new anti-mouse FXIIIA 675–688 antibody, also preimmune serum was used as a negative control. For colocalization, controls included staining for each primary antibody separately to exclude any cross reactivity between the two antibodies used. Slides were mounted with Gold anti-Fade medium (Invitrogen), dried overnight, and observed with a Leica DM IL inverted fluorescent microscope or by with a Leica DM 4000 B upright fluorescence microscope equipped with the Volocity image acquisition software (PerkinElmer, Toronto, ON). Black and white high-contrast images were generated from initial color images using Adobe Photoshop; images were subjected to brightness and contrast manipulation only.

### Statistical analysis

Experimental error is expressed as standard error of the mean (s.e.m.) of three independent experiments. Statistical significance was assessed by applying Student's *T*-test using Microsoft Excel software. *P* values are as follows: *p>0.1, **p>0.001, ***p>0.001.

## Supporting Information

Figure S1 **Effect of control compound NC10 on osteoblast collagen deposition in osteoblast cultures.** (A) Structre of NC10. (B) Picrosirious staining of COL I in cultures treated with either inhibitor NC9 or control NC10. COL I remained at similar levels as in controls with NC10. (C) Western blot analysis using anti-dansyl antibody. No dansyl group was observed to be incorporated into any proteins in cultures grown in the presence of NC10.
(TIF)

Figure S2 **FXIIIA antibody (Ab676) validation.** (A)(B) Immunofluoresence and RT-PCR detection of FXIIIA in medium only treated and differentiating MC3T3-E1 osteoblasts. Nondifferentiating cells express very low levels of FXIIIA and do not show any cellular FXIIIA staining with rabbit polyclonal antibody Ab676. (C) FXIIIA knockout osteoblasts were isolated from mouse calvariae according to a previously reported methods [101] and grown in culture for 10 days. Proteins were extracted and FXIIIA was detected by Western blotting. Rabbit polyclonal antibody Ab675 is shown to detect full length FXIIIA in wild type (WT) cells but not in FXIIIA deficient osteoblasts.
(TIF)

Figure S3 **Immunofluoresence staining of cytosolic GAPDH and nuclear stain DAPI in nonpermeabilized and permeabilized cells.** Staining patters show that permeabilization allows visualization of cytosolic protein (GAPDH) where as omission of this step block this staining. DAPI staining is visible with both techniques.
(TIF)

Figure S4 **Effect of NC9 on MTs in cells that do not express FXIIIA.** Tubulin staining in nonpermeabilized MC3T3-E1 osteoblasts grown with medium only and in the presence and absence of NC9. Figure S3 shows that these cells do not express FXIIIA. Staining patterns show that patchy MT network on the plasma membrane is not affected.
(TIF)
