# Supplementary figures and images for: Plasma Membrane Factor XIIIA Transglutaminase Activity Regulates Osteoblast Matrix Secretion and Deposition by Affecting Microtubule Dynamics
Source: PLoS One. 2011 Jan 20;6(1):e15893. doi: 10.1371/journal.pone.0015893 (PMC3024320; doi:10.1371/journal.pone.0015893)

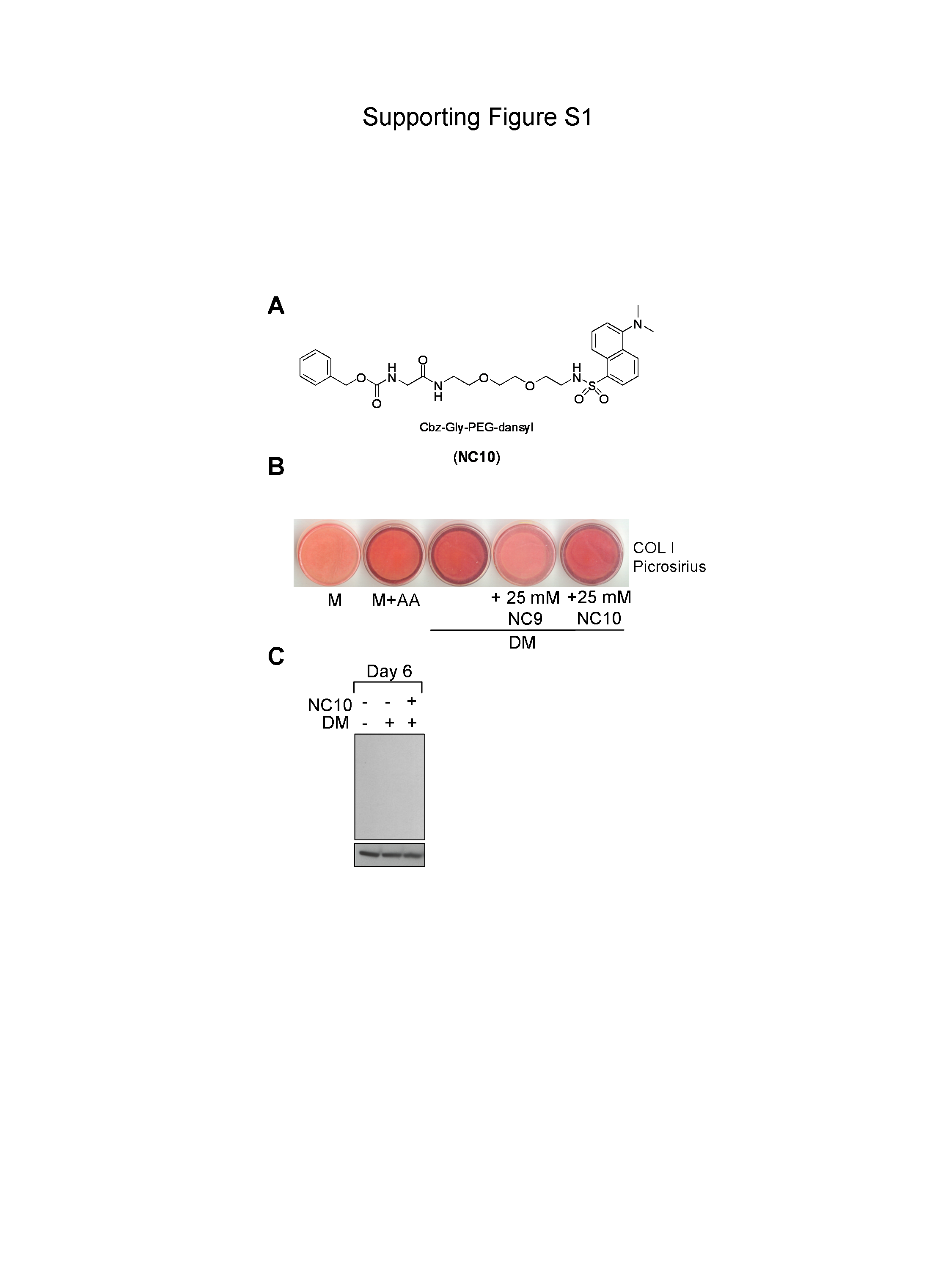

Supplement: Figure S1 — Effect of control compound NC10 on osteoblast collagen deposition in osteoblast cultures. (A) Structre of NC10. (B) Picrosirious staining of COL I in cultures treated with either inhibitor NC9 or control NC10. COL I remained at similar levels as in controls with NC10. (C) Western blot analysis using anti-dansyl antibody. No dansyl group was observed to be incorporated into any proteins in cultures grown in the presence of NC10. (TIF) [file pone.0015893.s001.tif]

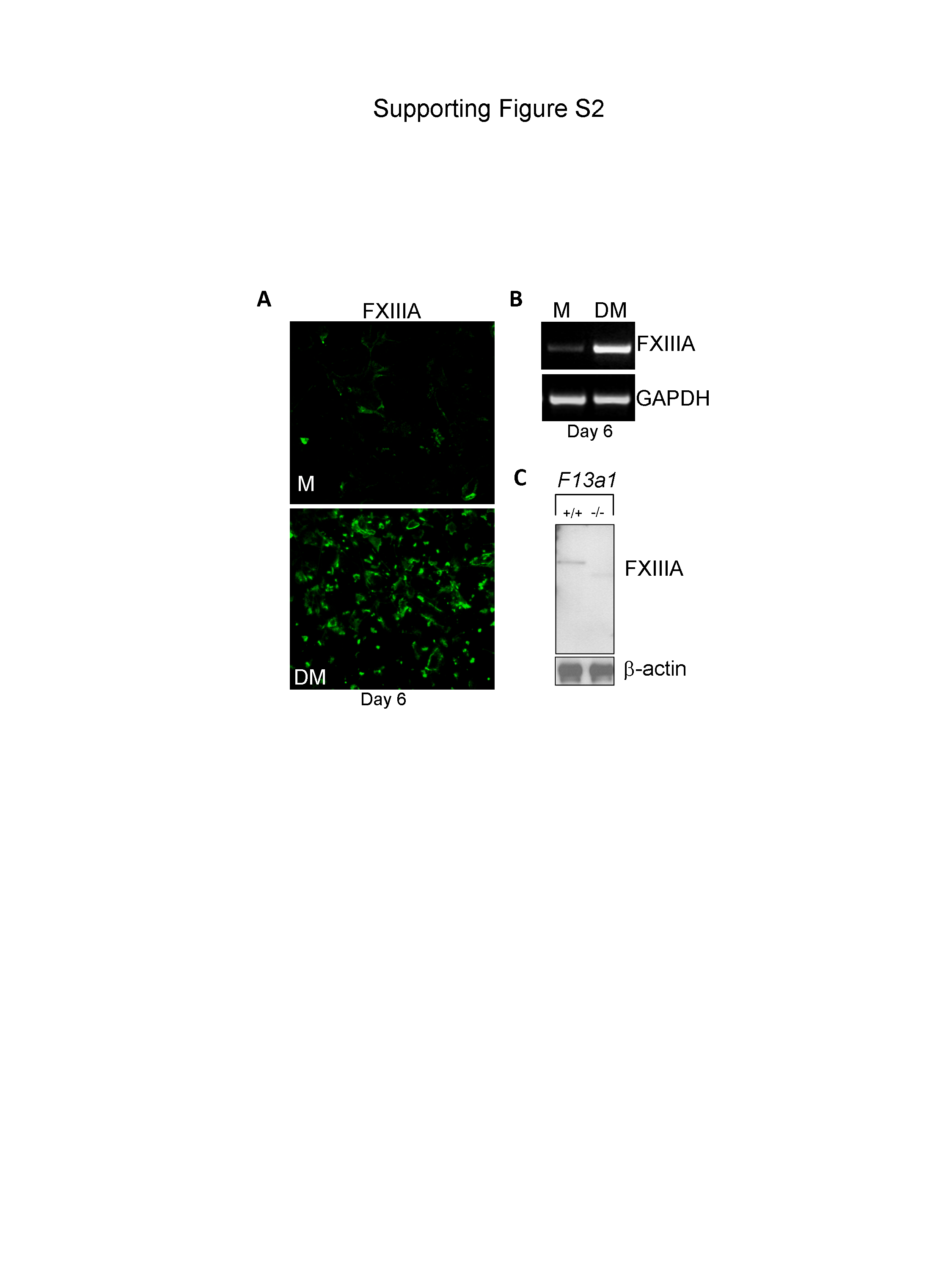

Supplement: Figure S2 — FXIIIA antibody (Ab676) validation. (A)(B) Immunofluoresence and RT-PCR detection of FXIIIA in medium only treated and differentiating MC3T3-E1 osteoblasts. Nondifferentiating cells express very low levels of FXIIIA and do not show any cellular FXIIIA staining with rabbit polyclonal antibody Ab676. (C) FXIIIA knockout osteoblasts were isolated from mouse calvariae according to a previously reported methods [101] and grown in culture for 10 days. Proteins were extracted and FXIIIA was detected by Western blotting. Rabbit polyclonal antibody Ab675 is shown to detect full length FXIIIA in wild type (WT) cells but not in FXIIIA deficient osteoblasts. (TIF) [file pone.0015893.s002.tif]

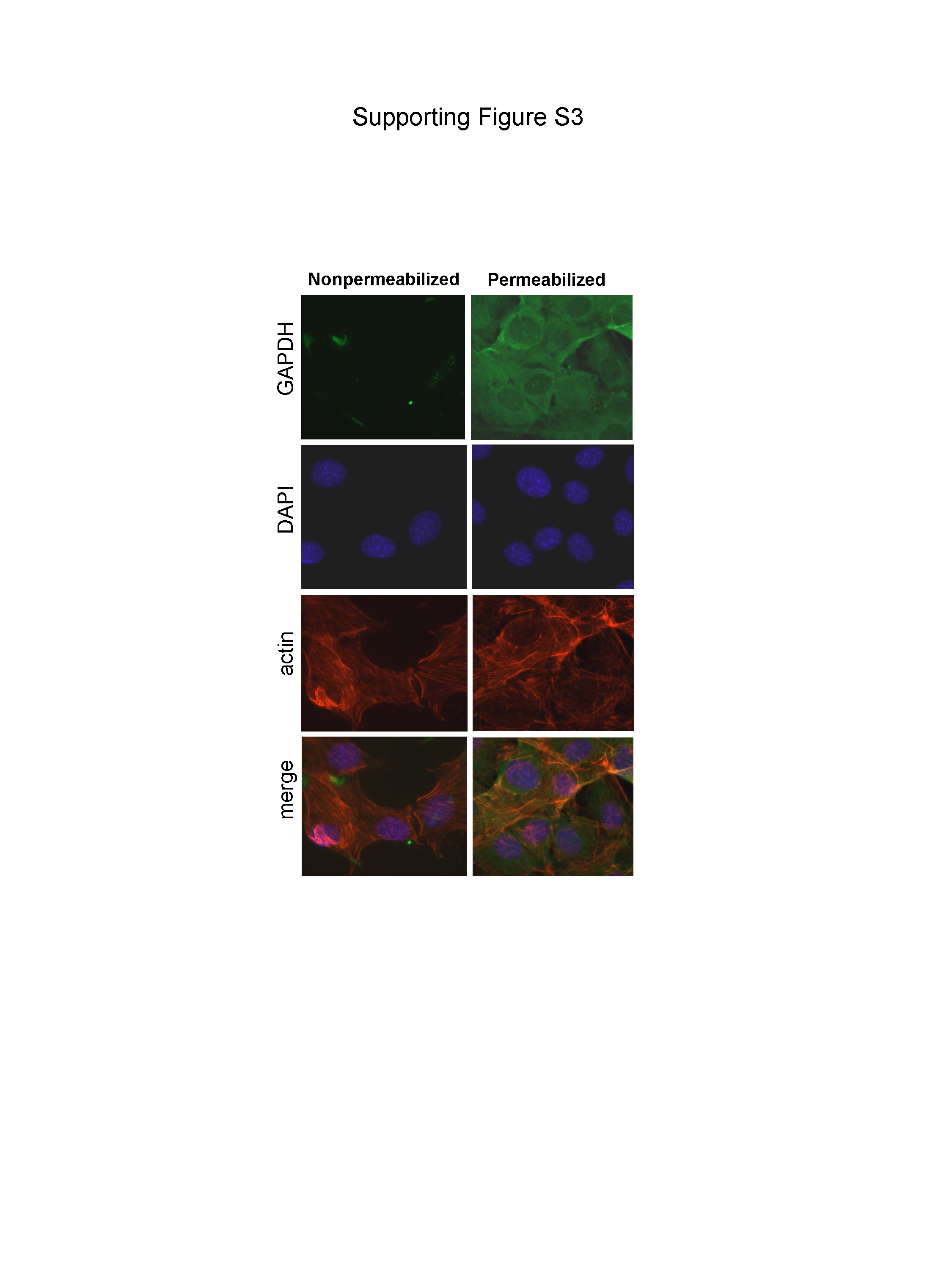

Supplement: Figure S3 — Immunofluoresence staining of cytosolic GAPDH and nuclear stain DAPI in nonpermeabilized and permeabilized cells. Staining patters show that permeabilization allows visualization of cytosolic protein (GAPDH) where as omission of this step block this staining. DAPI staining is visible with both techniques. (TIF) [file pone.0015893.s003.tif]

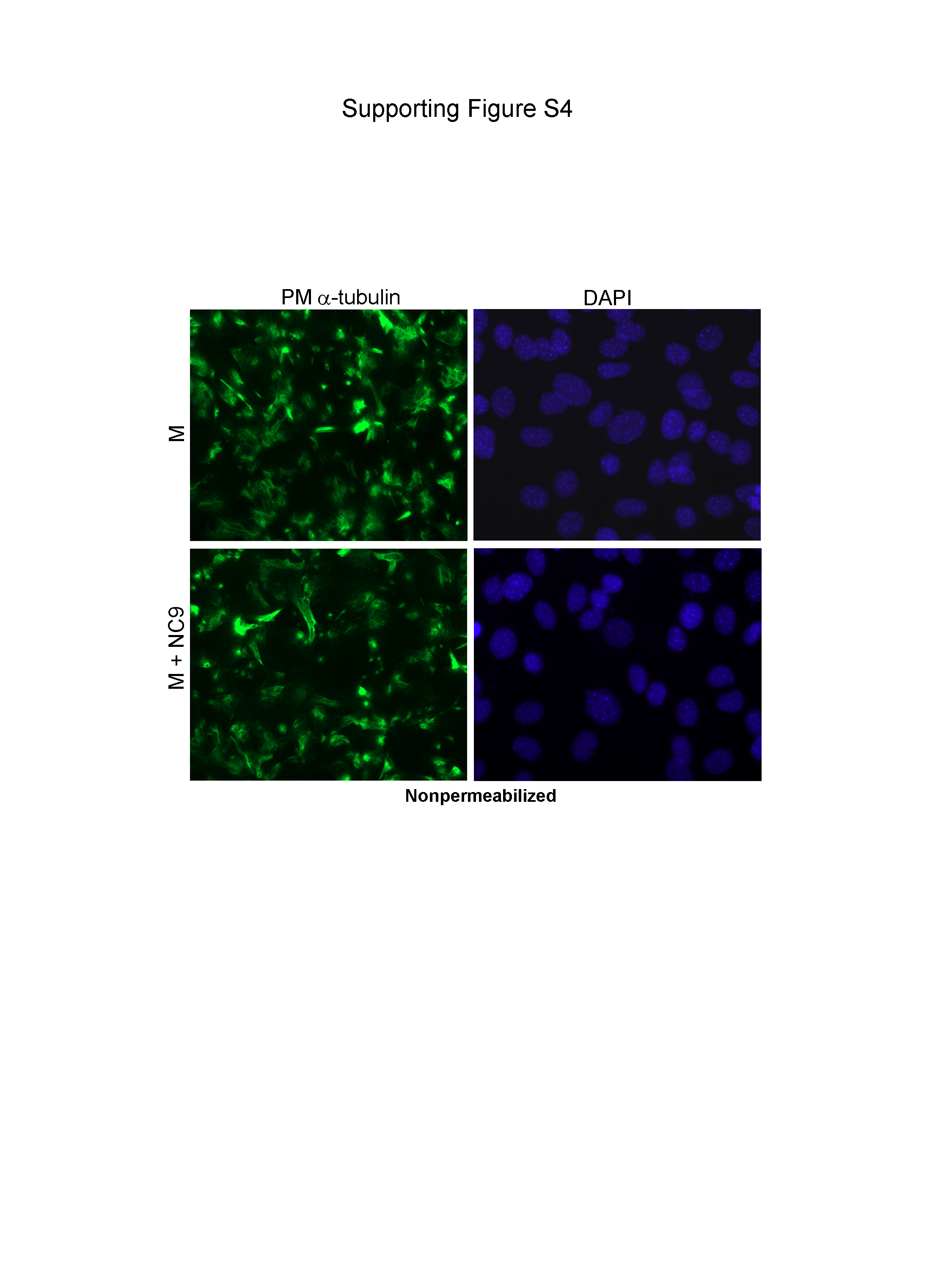

Supplement: Figure S4 — Effect of NC9 on MTs in cells that do not express FXIIIA. Tubulin staining in nonpermeabilized MC3T3-E1 osteoblasts grown with medium only and in the presence and absence of NC9. Figure S3 shows that these cells do not express FXIIIA. Staining patterns show that patchy MT network on the plasma membrane is not affected. (TIF) [file pone.0015893.s004.tif]
